# Partial Identification of Latent Correlations with Ordinal Data

**DOI:** 10.1007/s11336-022-09898-y

**Published:** 2023-01-31

**Authors:** Jonas Moss, Steffen Grønneberg

**Affiliations:** 1 Department of Data Science and Analytics, BI Norwegian Business School, 0484 Oslo, Norway; 2 Department of Economics, BI Norwegian Business School, 0484 Oslo, Norway

**Keywords:** polychoric correlation, partial identification, ordinal data

## Abstract

The polychoric correlation is a popular measure of association for ordinal data. It estimates a latent correlation, i.e., the correlation of a latent vector. This vector is assumed to be bivariate normal, an assumption that cannot always be justified. When bivariate normality does not hold, the polychoric correlation will not necessarily approximate the true latent correlation, even when the observed variables have many categories. We calculate the sets of possible values of the latent correlation when latent bivariate normality is not necessarily true, but at least the latent marginals are known. The resulting sets are called partial identification sets, and are shown to shrink to the true latent correlation as the number of categories increase. Moreover, we investigate partial identification under the additional assumption that the latent copula is symmetric, and calculate the partial identification set when one variable is ordinal and another is continuous. We show that little can be said about latent correlations, unless we have impractically many categories or we know a great deal about the distribution of the latent vector. An open-source R package is available for applying our results.

**Supplementary Information:**

The online version contains supplementary material available at 10.1007/s11336-022-09898-y.

The empirical covariance matrix for continuous data is consistent and asymptotically normal, enabling the use of a single asymptotic framework for inference in structural equation models (Browne, 1984; Satorra, 1989). But with ordinal data, the situation is more complex.

When the data is a random sample of vector variables with ordinal coordinates, it is usually inappropriate to estimate structural equation models directly on the covariance matrix of the observations (Bollen, 1989, Chapter 9). Instead, the correlation matrix of a latent continuous random vector *Z* is used as input for the models, such as ordinal factor analysis (Christoffersson, 1975; Muthén, 1978), ordinal principal component analysis (Kolenikov & Angeles, 2009), ordinal structural equation models (Jöreskog, 1984; Muthén, 1994), and, more recently, ordinal methods in network psychometrics (Epskamp, 2017; Isvoranu & Epskamp, 2021; Johal & Rhemtulla, 2021).

The polychoric correlation (Olsson, 1979) is the correlation of a latent bivariate normal variable based on ordinal data. While the polychoric correlation is an important dependency measure for ordinal variables under the bivariate normality assumption, its prime application lies in empirical psychometrics. In particular, it is employed in the two-stage estimation method for ordinal factor analysis and ordinal structural equation models. To employ the two-stage method, first estimate the latent correlation matrix using polychoric correlations, then fit a covariance model to this correlation matrix (Jöreskog, 2005). The method is implemented in current software packages such as EQS (Bentler, 2006), Mplus (Muthén & Muthén, 2012), LISREL (Jöreskog & Sörbom, 2015), and lavaan (Rosseel, 2012), and is frequently employed by researchers.

The polychoric correlation is guaranteed to equal the true latent correlation only if the continuous latent vector is bivariate normal, and is not, in general, robust against non-normality (Foldnes & Grønneberg, 2019b, a). Moreover, the inconsistent estimates of the latent correlation are transferred to ordinal structural equation models (Foldnes & Grønneberg, 2021). Multivariate normality has some testable implications (Foldnes & Grønneberg, 2019b; Jöreskog, 2006; Maydeu-Olivares, 2019b), and empirical datasets are frequently incompatible with it (Foldnes & Grønneberg, 2022). It is therefore important to consider what can be said about the latent correlations that can generate an observed ordinal variable under weaker conditions than bivariate normality.

This paper continues Grønneberg, Moss, and Foldnes (2020) in calculating the possible values of a latent correlation when knowing only the marginal distributions of the latent variable, but not its copula. This type of calculation is called partial identification analysis (Manski, 2010; Tamer, 2003). While Grønneberg et al. (2020) studied binary data, we study ordinal data with an arbitrary number of categories. As in Grønneberg et al. (2020), our analysis is at the population level. Inference for partial identification sets can be done using the methods of Tamer (2010, Section 4.4). Our partial identification analyses are done for a single latent correlation only, even though the multivariate setting is of greater psychometric interest. Simultaneous partial identification sets for the covariance matrix will be difficult to calculate, as even the set of $3\times 3$ correlation matrices without any restrictions is hard to describe (Li & Tam, 1994).

Let *Z* be a bivariate continuous latent variable with correlation $\rho$, which we call the latent correlation. We are dealing with ordinal variables $(X,Y)$ with $I, J$ categories generated via the equations

1 $$\begin{aligned} X= {\left\{ \begin{array}{ll} 1, &{} \text {if } Z_1 \le \tau ^X_{1} \\ 2, &{} \text {if } \tau ^X_{1}< Z_1 \le \tau ^X_{2} \\ \vdots &{} \\ I, &{} \text {if } \tau ^X_{(I-1)}< Z_1 \\ \end{array}\right. } \quad \quad Y= {\left\{ \begin{array}{ll} 1, &{} \text {if } Z_2 \le \tau ^Y_{1} \\ 2, &{} \text {if } \tau ^Y_{2}< Z_2 \le \tau ^Y_{3} \\ \vdots &{} \\ J, &{} \text {if } \tau ^Y_{(J-1)} < Z_2 \\ \end{array}\right. } \end{aligned}$$

where $\tau ^X\in \mathbb {R}^{I-1}$ and $\tau ^Y\in \mathbb {R}^{J-1}$ are strictly increasing vectors of deterministic thresholds. Our goal is to identify the possible values of the latent correlation $\rho$ from the distribution of the latent variable, plus potentially some more information.

We will show that knowing only the marginals of *Z* is insufficient for pinpointing the latent correlation to high precision, even when the number of categories is as high as ten. High precision can only be achieved by making assumptions about the copula of the latent variable as well. We calculate the set of possible values of the latent variable when the copula of the latent variable is known to be symmetric and its marginals are known. While this reduces the range of the possible values of the latent variable, the reduction is small. We also study partial identification of $\rho$ when $Z_2$ is directly observed, i.e., the polyserial correlation (Olsson, Drasgow, & Dorans, 1982) without assuming bivariate normality. Methods for calculating the resulting bounds on the latent correlations are implemented in the R package polyiden available in the online supplementary material and on Github^1^.

The core results of this paper generalize the results in Grønneberg et al. (2020) from two categories to an arbitrary number of categories. Our emphasis is on aspects that appear when $I$ or $J$ is higher than 2, such as asymptotic results when $I$ or $J$ increase separately. We show that when the marginal distributions of the latent variable are known, the latent correlation is asymptotically identified when both $I$ and $J$ increase. Moreover, when only $J$ increases, the identification region of $\rho$ approaches the identification region found when one variable is directly observed.

We consider the case where the copula of the latent variables is completely unknown (or known to be symmetric) except for the restrictions given from the distribution of the observations. As argued above, additional assumptions on the copula are needed to better pinpoint the latent correlation. One possibility is to consider a parametric class of copulas, and identify the set of possible Pearson correlations compatible with this class. Another possibility is to consider stronger but still nonparametric assumptions, such ellipticity. Such additional assumptions would lead to shorter partial identification sets than those we find, but their calculation is outside the scope of the paper.

There are several alternative ways of formulating psychometric models for ordinal data that are not dependent on latent correlations, the most prominent being variants of item response theory (see, e.g.,  Bartholomew, Steele, Galbraith, & Moustaki, 2008). While a large class of commonly used item response theory models are mathematically equivalent to ordinal covariance models (Foldnes & Grønneberg, 1987; Takane & De Leeuw, 2019a), the models are usually estimated directly in terms of the model parameters using maximum likelihood or Bayesian methods (Van der Linden, 2017, Section III). These models are usually conceptualized in fully parametric terms, so our analysis is less relevant for such models.

In cases where the dimensionality of the item response theory model is unknown, i.e., the model is not fully specified in terms of continuously varying parameters, a factor analysis based on polychoric correlations is sometimes recommended, see, e.g., Mair (2018, Section 4.1.2), Brown and Croudace (2014, p. 316), Revicki, Chen, and Tucker (2014, p. 344), Zumbo (2006, Section 3.1). From this perspective, our work also has relevance for item response theory models.

Recently, structural equation models based on copulas have been suggested (Krupskii & Joe, 2013, 2015), and Nikoloulopoulos and Joe (2015) deals specifically with copula motivated models for ordinal data. Since we focus specifically on correlations, our analysis is not relevant for such models.

We focus exclusively on the Pearson correlation of the latent continuous vector *Z*, and do not consider the more general problem of quantifying and analyzing dependence between discrete variables. Several papers have been written in this more general direction. For instance, Liu, Li, Yu, and Moustaki (2021) introduces partial association measures between ordinal variables, Nešlehová (2007) discuss rank correlation measures for non-continuous variables, and Wei and Kim (2017) introduces a measure for asymmetric association for two-way contingency tables. Constraints on concordance measures in bivariate discrete data are derived in Denuit and Lambert (2005). Finally, we mention the multilinear extension copula discussed in Genest, Nešlehová, and Rémillard (2014); Genest, Nešlehová, and Rémillard (2017) which provides an abstract inference framework for a large class of copula based empirical methods for count data.

The structure of the paper is as follows. We start by studying partial identification sets for latent correlations based on ordinal variables in Sect. 1. Then, in Sect. 2, we study the same problem, but allow $Z_2$ to be directly observed. In Sect. 3 we illustrate the results with a detailed example, and Sect. 4 concludes the paper. All proofs and technical details are in the online appendix, including a short introduction to copulas. Scripts in R (R Core Team, 2020) for numerical computations are available in the online supplementary material.

## Latent Correlations on $I\times J$ Tables

We work with the distribution function of the ordinal variable $(X, Y)$, which can be described by the *cumulative probability matrix* ${\varvec{\Pi }}$ with elements ${\varvec{\Pi }}_{ij} = P(X\le i,Y\le j)$ for $i = 1,\ldots ,I,\;j= 1,\ldots ,J$. The model for $(X, Y)$ follows the discretization model defined in eq. (1) for some continuous *Z* with marginal distribution functions $F_1,F_2$.

Observe that

2 $$\begin{aligned} {\varvec{\Pi }}_{ij} = P(F_1(Z_1) \le {\varvec{\Pi }}_{iJ}, F_2(Z_2) \le {\varvec{\Pi }}_{Ij}) = C({\varvec{\Pi }}_{iJ}, {\varvec{\Pi }}_{Ij}) \end{aligned}$$

where *C* is the *copula* of *Z* (see, e.g., Nelsen, 2007). It follows that the copula *C* restricted to $A = \{{\varvec{\Pi }}_{iJ},i=1,\cdots , I\}\times \{{\varvec{\Pi }}_{Ij} \mid j = 1,\cdots , J\}$ encodes all available information about *Z*. Since *A* is a product set with both factors containing 0 and 1, the restriction of *C* to *A* is a *subcopula* of *C* (Carley, 2002).

Now we are ready to state our first result.

### Proposition 1

For any cumulative probability matrix ${\varvec{\Pi }}$, the latent correlation can be any number in $(-1,1)$ when the marginals $F_{1}$ and $F_{2}$ are unrestricted.

### Proof

See the online appendix, Section 8. $\square$

Proposition 1 implies that we have to know something about the marginals $F_1, F_2$ to get non-trivial partial identification sets for the latent correlation. Now we consider the case when both marginals are known. Let $\mathcal {F}$ be a set of bivariate distribution functions, and $\rho (F)$ be the Pearson correlation for a bivariate distribution *F*. Define the partial identification set for the latent correlation as

3 $$\begin{aligned} \rho _{\varvec{\Pi }}(\mathcal {F}) = \left\{ \rho (F)\mid F\text { is compatible with }{\varvec{\Pi }}\text { and }F\in \mathcal {F}\right\} , \end{aligned}$$

where *F* is compatible with ${\varvec{\Pi }}$ if equation (2) holds for its copula.

Now define the $I\times J$ matrices $\alpha ,\beta ,\gamma ,\delta$ with elements

4 $$\begin{aligned} \alpha _{ij}&= {\varvec{\Pi }}_{(i-1)J}+{\varvec{\Pi }}_{i(j-1)}-{\varvec{\Pi }}_{(i-1)(j-1)} = P(X<i)+P(X=i,Y<j),\nonumber \\ \beta _{ij}&= {\varvec{\Pi }}_{I(j-1)}+{\varvec{\Pi }}_{(i-1)j}-{\varvec{\Pi }}_{(i-1)(j-1)} = P(Y<j)+P(X<i,Y=j),\nonumber \\ \gamma _{ij}&= {\varvec{\Pi }}_{iJ}-({\varvec{\Pi }}_{i(J-j+1)}-{\varvec{\Pi }}_{(i-1)(J-j+1)}) = P(X\le i)-P(X=i,Y\le J-j+1),\nonumber \\ \delta _{ij}&= {\varvec{\Pi }}_{I(J-j+1)}-({\varvec{\Pi }}_{i(J-j+1)}-{\varvec{\Pi }}_{i(J-j)}) = P(X\le J-j+1)-P(X\le i,Y=J-j+1). \end{aligned}$$

where ${\varvec{\Pi }}_{0j} = {\varvec{\Pi }}_{i0}=0$. Then define the vectorswhere $a\frown b$ is the concatenation of the vectors *a*, *b* and  is the vectorization of *A*, obtained from stacking the columns of *A* on top of each other. The matrices in (4) are the same as the $\alpha ,\beta ,\gamma ,\delta$ matrices of Genest and Nešlehová (2007, p. 481) and Carley (2002), only the order of $\gamma$ and $\delta$ has been changed. We have made this minor modification as it is needed to make $u^U$ and $u^L$ increasing, which simplifies the statement of the next result.

The following result extends Proposition 5 in Genest and Nešlehová (2007), who built their result on the work of Carley (2002) on maximal extensions of subcopulas, to the case of non-uniform marginals.

### Theorem 1

Let $\mathcal {F}$ be the set of distributions with continuous and strictly increasing marginals $F_1, F_2$ with finite variance. Then $\rho _{\varvec{\Pi }}(\mathcal {F}) = [\rho _L, \rho _U]$ where

5 $$\begin{aligned} \rho _U= & {} {\text {sd}} (F_1)^ {-1} {\text {sd}} (F_2)^ {-1} \left( \sum _{k=1}^{IJ}\int _{u_{k}^{U}}^{u^U_{k+1}}F_{1}^{-1}(u)F_{2}^{-1}(v_{k}^U-u^U_{k}+u)du-\mu _{F_{1}}\mu _{F_{2}} \right) , \end{aligned}$$

6 $$\begin{aligned} \rho _L= & {} {\text {sd}} (F_1)^ {-1} {\text {sd}} (F_2)^ {-1} \left( \sum _{k=1}^{IJ}\int _{u_{k}^{L}}^{u_{k+1}^{L}}F_{1}^{-1}(u)F_{2}^{-1}(v_{k}^{L}+u_{k+1}^{L}-u)du-\mu _{F_{1}}\mu _{F_{2}} \right) , \end{aligned}$$

where $F_{1}^{-1}$ and $F_{2}^{-1}$ are the generalized inverses of $F_{1},F_{2}$, where $\mu _{F_{1}},\mu _{F_{2}}$ are the means of $F_{1}$ and $F_{2}$, and where ${\text {sd}} (F_1), {\text {sd}} (F_2)$ are the standard deviations of $F_1,F_2$.

### Proof

See the online appendix, Section 11. $\square$

### Example 1

Let us compute the partial identification limits in Theorem 1 for a sequence of cumulative probability matrices. Let $Z$ have a bivariate normal copula with correlation $\rho = 0.7$. We study what an analyst who does not know the copula structure of $Z$ can say about $\rho$.

To generate thresholds that plausibly fit real world settings and can be applied for any number of categories, we fit a statistical model to the marginal probability distribution of the bfi dataset from the psych package, a dataset described in more detail in Sect. 3. We estimated the parameters of a Beta distribution that best correspond to the ordinal marginals of the questions A2 and A5 using a least squares procedure; see the code for details. While the bfi dataset has six categories, we can emulate the marginal probabilities for any number of categories by choosing cutoffs $({\varvec{\Pi }}_{iJ})_{i=1}^I$ and $({\varvec{\Pi }}_{Ij})_{j=1}^J$ as follows. The cutoffs for $X$ with *k* categories are equal to $Q_1(i/k)$, $i = 1,\ldots ,(k-1)$, where $Q_1$ is the quantile function of a Beta distributed variable with parameters $\alpha _1 = 2.7, \beta _2 = 1.1$. The cutoffs for $Y$ are generated in the same way, but with $\alpha _2 = 2.3, \beta _2 = 1.2$.

**Fig. 1:**
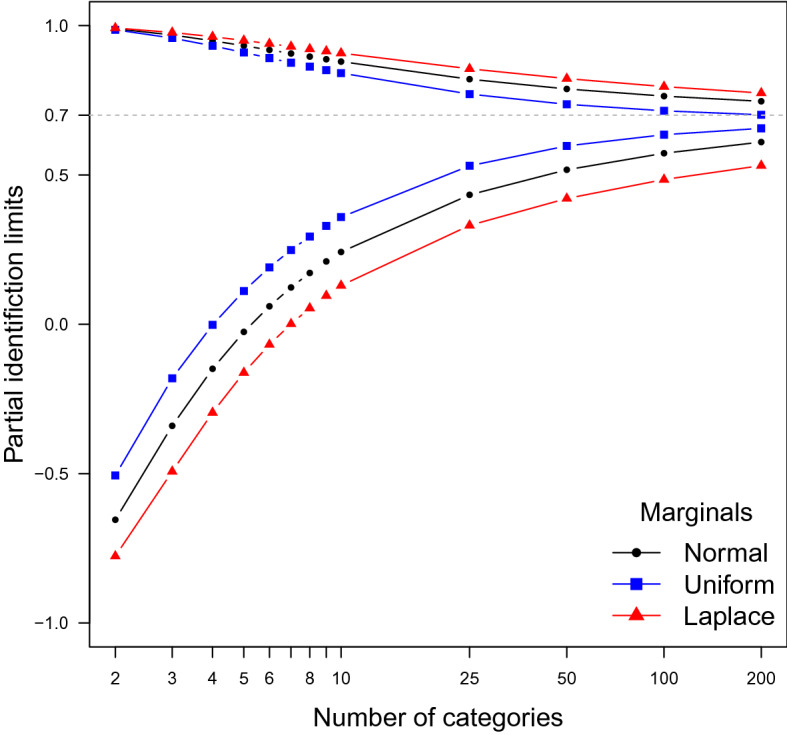
Upper and lower limits for $\rho _{\varvec{\Pi }}(\mathcal {F})$ when the marginals are fixed. The dashed line is the polychoric correlation, corresponding to normal marginals and the normal copula.

In Fig. 1, we see the partial identification region as a function of *I*, *J* when $I = J$. The latent marginals are either standard normal, standard Laplace distributed, or uniform on [0, 1]. The dotted line is the latent correlation ($\rho = 0.7$) when the marginals are normal. The true latent correlations are 0.682 when the marginals are uniform and 0.686 when the marginals are Laplace distributed. $\square$

Figure 1 suggests two conclusions. First, when the latent copula is completely unknown the identification sets are too wide to be informative even for a large number of categories, such as $I= J= 10$. Second, the partial correlation sets converge to the true latent correlations as the number of categories go to infinity. This is indeed the case when the marginals are known, as shown by the following corollary.

Consider a sequence $({\varvec{\Pi }}^n)_{n=1}^\infty$ of cumulative probability matrices where ${\varvec{\Pi }}^n$ has $I_n, J_n$ categories. We say that the sequence $({\varvec{\Pi }}^n)$ has its $X$-mesh uniformly decreasing to 0 if

7 $$\begin{aligned} x_n := \max _{1 \le i \le I_n}{[{\varvec{\Pi }}^n_{iJ_n} - {\varvec{\Pi }}^n_{(i-1)J_n}]} \rightarrow 0, \end{aligned}$$

and, likewise, its $Y$-mesh is uniformly decreasing to 0 if

8 $$\begin{aligned} y_n := \max _{1 \le j \le J_n}{[{\varvec{\Pi }}^n_{I_nj} - {\varvec{\Pi }}^n_{I_n(j-1)}]} \rightarrow 0. \end{aligned}$$

For a copula *C* and marginals $F_1, F_2$, let $\rho (C;F_1,F_2)$ be the Pearson correlation of the combined distribution $(x_1,x_2) \mapsto C(F_1(x_1), F_2(x_2))$.

### Corollary 1

Let $\mathcal {F}$ be the set of distributions with continuous and strictly increasing marginals $F_1, F_2$ and $({\varvec{\Pi }}^n)$ be a sequence of cumulative probability matrices compatible with *C* whose $X$-mesh and $Y$-mesh uniformly decrease to 0. Then the latent correlation identification set converges to $\rho (C;F_1,F_2)$, i.e., $\lim _{n\rightarrow \infty } \rho _{{\varvec{\Pi }}^n}(\mathcal {F}) = \rho (C;F_1,F_2)$.

### Proof

See the online appendix, Section 12. $\square$

Figure 1 illustrates Corollary 1. The sequence of ordinal distributions has uniformly decreasing *X*-mesh and *Y*-mesh, and the partial identification sets for normal marginals clearly converge to the true correlation as $n\rightarrow \infty$.

In Theorem 1, the latent marginals are fixed, and the latent copula is unknown. The numerical illustration in Fig. 1 shows that, even with a large number of categories such as ten, the partial identification intervals for latent correlations are rather wide. If our goal is to make the intervals shorter, we will have to add some restrictions to the copula. In Section 10 in the online appendix, we conduct a partial identification analysis based on the assumption that the latent copula is *symmetric* (Nelsen, 2007, p. 32). Unfortunately, symmetry does not shorten the identification intervals by much. More work is needed to find tractable restrictions on the copula that make the identification intervals shorter.

## Latent Correlations with One Ordinal Variable

Until now, we have studied the case where we could observe neither $Z_1$ nor $Z_2$. Now we take a look at the case when we are able to observe one of them. That is, we still observe the ordinal $X$ from the discretization model of equation  (1) but now we also observe the continuous $Z_2$. We are still interested in the correlation between the latent $Z_1$ and the now observed $Z_2$. Mirroring the fully ordinal case, the latent correlation is identified when $(Z_1, Z_2)$ is bivariate normal, and can be estimated by the polyserial correlation (Olsson et al., 1982). As before, the latent variable has known marginals $F_1,F_2$ but unknown copula *C*. Again, the latent correlation $\rho$ is not identified, and we find the partial identification set.

Assume that $F_2$ is continuous and strictly increasing, which implies that $V = F_2(Z_2)$ is uniformly distributed. Let ${\varvec{\Pi }}^\star$ be the cumulative distribution of $(X, V)$, that is, ${\varvec{\Pi }}^\star _{iv} = P(X\le i, V \le v)$. If *C* is the copula of *Z*, we get the relationship

9 $$\begin{aligned} {\varvec{\Pi }}^\star _{iv} = C({\varvec{\Pi }}^\star _{i1}, {\varvec{\Pi }}^\star _{Iv}) = C({\varvec{\Pi }}^\star _{i1}, v),\quad 1\le i \le I-1, v\in [0, 1]. \end{aligned}$$

Whenever *C* is a copula that satisfies the equation above, we say that *C* *is compatible with* ${\varvec{\Pi }}^\star$. From the results of Tankov (2011), we can derive the maximal and minimal copula bounds for every *C* satisfying Eq. (9). Using these bounds, we can derive the following result, which generalizes Proposition 3 in Grønneberg et al. (2020). We use the notation $x^+ = \max (x,0)$ and $x^- = \min (x,0)$.

### Theorem 2

Let $F_1, F_2$ be continuous and strictly increasing with finite variance, and let $\mathcal {F}$ be the set of distributions with marginals $F_1$ and $F_2$. Then the set of latent correlations that is compatible with $C,F_1,F_2$ is

10 $$\begin{aligned} \rho _{{\varvec{\Pi }}^\star }(\mathcal {F}) = [\rho (W_{{\varvec{\Pi }}^\star };F_1,F_2), \rho (M_{{\varvec{\Pi }}^\star };F_1,F_2)], \end{aligned}$$

where

$$\begin{aligned} M_{{\varvec{\Pi }}^\star }(u,v)&= \min (u,v,\min _{1\le i\le I-1}({\varvec{\Pi }}^\star _{i v}+(u-{\varvec{\Pi }}^\star _{i1})^{+}),\\ W_{{\varvec{\Pi }}^\star }(u,v)&= \max (0,u+v-1,\max _{1\le i\le I-1}({\varvec{\Pi }}^\star _{i v}-({\varvec{\Pi }}^\star _{i1}-u)^{+}). \end{aligned}$$

### Proof

See the online appendix, Section 13. $\square$

### Remark 1

To calculate the correlation $\rho (C,F_1,F_2)$, one may use the Höffding (1940) formula,

11 $$\begin{aligned} \rho (C;F_1,F_2) = {\text {sd}} (F_1)^ {-1} {\text {sd}} (F_2)^ {-1} \int _{0}^{1} \int _{0}^{1}\left[ C(u, v) - uv\right] \, \textrm{d}F_{1}^{-1}(u) \textrm{d}F_{2}^{-1}(v), \end{aligned}$$

where ${\text {sd}} (F_1), {\text {sd}} (F_2)$ are the standard deviations of $F_1,F_2$.

Let $({\varvec{\Pi }}^n)_{n=1}^\infty$ be a sequence of cumulative probability matrices where $I_n = I\ge 2$ is fixed and $J_n \rightarrow \infty$. Then we ought to regain the polyserial identification set of Theorem 2 under reasonable assumptions. This is formalized and confirmed by the following corollary.

### Corollary 2

Let $({\varvec{\Pi }}^n)_{n=1}^\infty$ be a sequence of cumulative probability matrices compatible with *C*. Let $I$ be fixed for all *n* and let $J_n$ diverge to infinity, and let the *Y*-mesh of $({\varvec{\Pi }}^n)_{n=1}^\infty$ decrease uniformly toward 0. Let ${\varvec{\Pi }}^\star$ have $I$ categories and be compatible with *C*. If $\mathcal {F}$ is the set of distributions with continuous and strictly increasing marginal distributions $F_1, F_2$, then

$$\begin{aligned} \lim _{n\rightarrow \infty } \rho _{{\varvec{\Pi }}^n}(\mathcal {F}) = \rho _{{\varvec{\Pi }}^\star }(\mathcal {F}). \end{aligned}$$

### Proof

See the online appendix, Section 14. $\square$

**Fig. 2:**
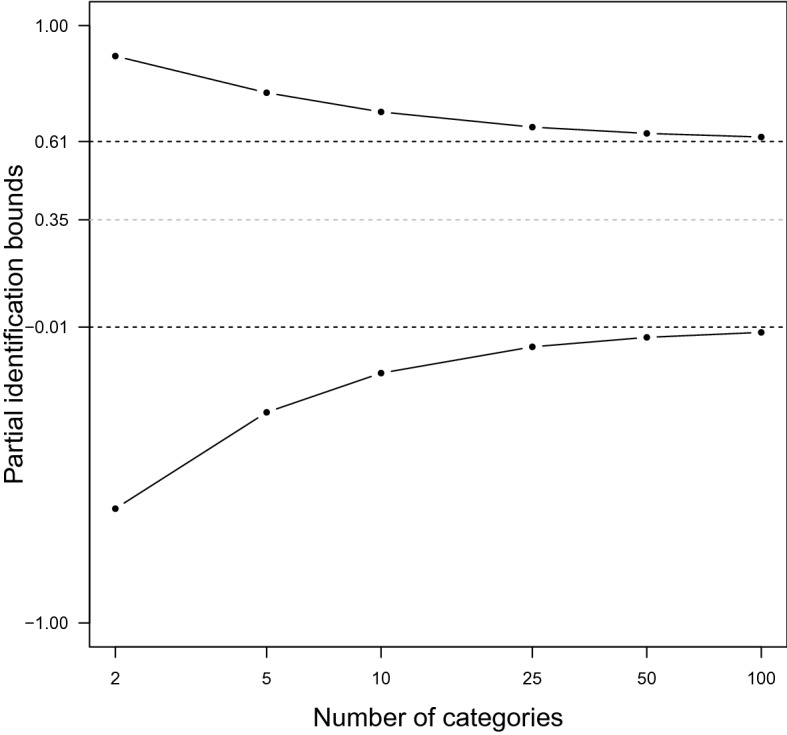
Illustration of Theorem 2 and Corollary 2. The black lines are the limits of the identification sets in Corollary 2, the black dashed lines are the limits of identification sets in Theorem 2, and the gray dashed line is the true polychoric correlation.

Theorem 2 and Corollary 2 are illustrated in Fig. 2. We use the same setup as Example 1 on p. 1. The marginals of $Z$ are normal and known to be so, the true copula is bivariate normal, but this is not known, and the true latent correlation is 0.35. The number of categories for $X$ is 4. The number of categories for $Y$ increase indefinitely, and the *Y*-mesh uniformly decreases toward 0. We used the polyserialiden function in the R package polyiden to calculate the polyserial bounds.

## An Empirical Example Using Data from the International Personality Item Pool

The R package psychTools (Revelle, 2019) contains the dataset bfi, which is a small subset of the data presented and analyzed in Revelle, Wilt, and Rosenthal (2010) based on items from the International Personality Item Pool (Goldberg, 1999). The bfi dataset contains 2800 responses to 25 items, and are organized by the five factors of personality: Agreeableness (A1–A5), Conscientiousness (C1–C5), Extraversion (E1–E5), Neuroticism (N1–N5), and Openness to experience (O1–O5). Each response is graded on a 6-point scale from “Very Inaccurate” to “Very Accurate.” A sample question is A2: “Inquire about others’ well-being.” We have flipped the ratings on reverse-coded items, and omitted all rows with missing values, resulting in 2236 remaining observations.

**Fig. 3:**
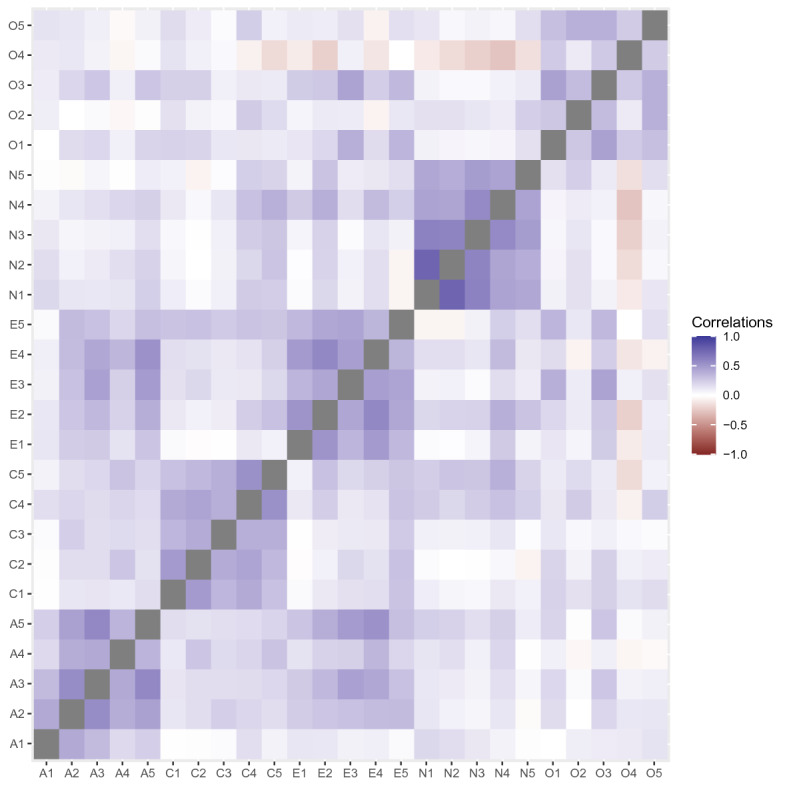
Polychoric correlation estimates for 25 items from the International Personality Item Pool (Goldberg, 1999).

The polychoric correlations are visualized in Fig. 3. This dataset has been used for illustrations in several contexts, for instance in the second empirical example of McNeish (2018), who analyzed the data using a five factor model estimated via polychoric correlations as well as Pearson correlations.

Polychoric correlations and models that use them for input in their estimation, hinges on the exact normality of each bivariate pair of the latent continuous variable $Z$. As shown theoretically in this paper, and through simulation in Foldnes and Grønneberg (2019b, 2021), polychoric correlations are not robust against latent non-normality. The assumption of joint normality has testable implications, and we applied the parametric bootstrap test of Foldnes and Grønneberg (2019b) using the R package discnorm (Foldnes & Grønneberg, 2020), which has been shown to behave well in the simulation studies of Foldnes and Grønneberg (2019b, 2021). We test both multivariate normality of the 25 dimensional random vector, as well as bivariate normality of each pair of variables. The resulting *p* values for the joint test of multivariate normality was zero within numerical precision. Out of 300 pairs, 184 pairs had a *p* value of latent normality also equal to zero within numerical precision, 287 pairs had a *p* value less than $5 \%$, and the mean of the *p* values equaled $0.99 \%$. Latent normality is therefore not a tenable assumption, with the possible exception of bivariate latent normality between some pairs of variables.

**Fig. 4:**
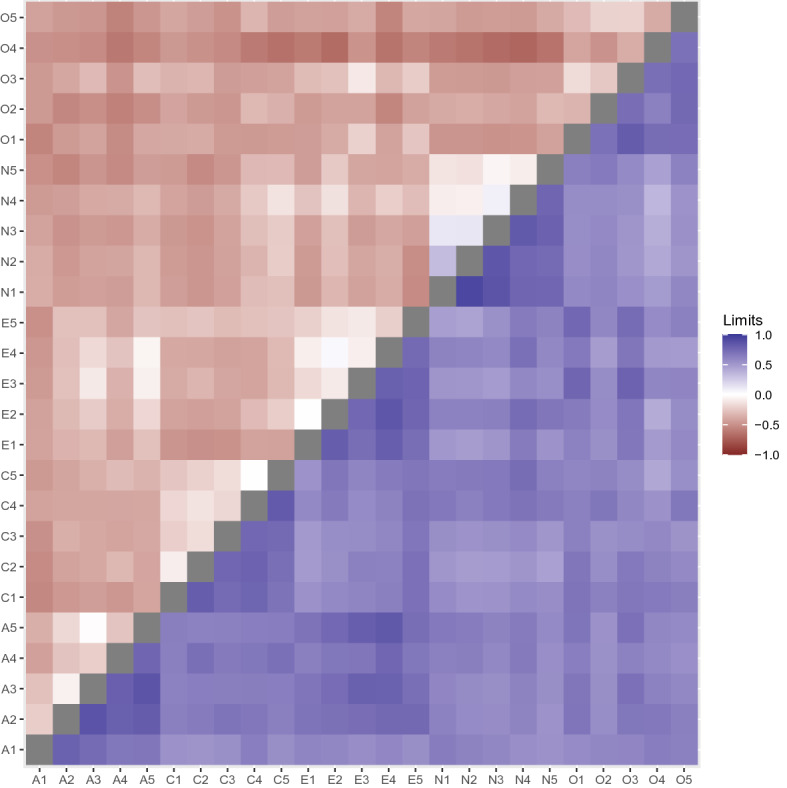
Upper (blue) and lower (red) correlation bounds for the items in the International Personality Item Pool (Goldberg, 1999).

We therefore calculate the lower and upper latent correlation bounds from Theorem 1, when assuming marginal but not bivariate normality. The results are visualized in Fig. 4. The bounds are large for all variables; the sign of the correlations are unknown in most cases, even in the same factor, though we see indications of white regions in the lower bounds (indicating lower bounds near zero) for the agreeableness items, the conscientiousness items, extroversion items, and neuroticism items, but not for the openness items. For neuroticism (N1–N5), most of the correlations are positive. There are some other pairs with lower bounds near zero, such as the bright region between A5 and E3–E4, where the lower bounds are near zero and the upper bounds are close to one, which under the assumption of latent marginal normality shows that the latent correlations between these items are estimated to be positive.

## Conclusion

We have calculated partial identification sets for latent correlations based on the distribution of ordinal data under the assumption that the marginal distributions of $Z$ are known. The most common number of categories is 5 and 7 (Flora & Curran, 2012; Rhemtulla, Brosseau-Liard, & Savalei, 2004), and for these numbers the partial identification sets are rather wide. Merely knowing the latent marginal distributions is usually insufficient, and knowing that the latent copula is symmetric does not help. More knowledge is required in order to get informative partial identification sets.

Since the partial identification sets are wide, a psychometrician wishing to estimate latent correlations must know more about the latent distribution class than just its marginals and possibly knowing that the copula is symmetric. For instance, the copula class could be known to belong to a certain class of distributions, or the psychometrician may know that $Z$ follows a model class, such as a factor model. Such knowledge would reduce the partial identification sets of the model parameters.

## Supplementary Information

Below is the link to the electronic supplementary material.

Supplementary file 1 (txt 0 KB)

Supplementary file 2 (r 1 KB)

Supplementary file 3 (rdata 46 KB)

Supplementary file 4 (r 0 KB)

Supplementary file 5 (r 0 KB)

Supplementary file 6 (r 3 KB)

Supplementary file 7 (r 3 KB)

Supplementary file 8 (r 1 KB)

Supplementary file 9 (r 1 KB)

Supplementary file 10 (pdf 420 KB)

Supplementary file 11 (r 6 KB)

Supplementary file 12 (zip 1097 KB)

## Footnotes

1 https://github.com/JonasMoss/polyiden
