# Supplementary material for: Partial Identification of Latent Correlations with Ordinal Data
Source: Psychometrika. 2023 Jan 31;88(1):241–52. doi: 10.1007/s11336-022-09898-y (PMC9977897; doi:10.1007/s11336-022-09898-y)
Supplement: Supplementary file 10 — (pdf 420 KB) [file 11336_2022_9898_MOESM10_ESM.pdf]

ONLINE SUPPLEMENTARY MATERIAL: APPENDIX

Jonas Moss and Steffen Grønneberg

This online appendix starts with Section 7 which gives basic definitions and results for copulas. We continue with Section 8, where we prove some results on shuffles of min copulas, as defined in Mikusinski, Sherwood, and Taylor (1992). In Section 9, we provide a general framework for partial identification for Pearson correlations based on restrictions on the copula, where the restriction set is a compact product set. These results are based on improved Fréchet–Höffding bounds from Bernard, Liu, MacGillivray, and Zhang (2013); Tankov (2011). The general framework is used several places in the paper. We then move on to an analysis of partial identification under the additional restriction of a symmetric copula in Section 10. Finally, all remaining proofs are given in separate sections.

7. Definitions and basic copula results

We start out with a characterization of cumulative probability matrices. A well-known property of discrete distributions is that they are valid distributions if and only if the probability mass function sums to one and is contained in $[0,1]$. The following proposition translates this property to cumulative probability matrices.

Proposition 2. *Let* $\mathbf{\Pi}$ *be a* $I \times J$ *matrix. Then* $\mathbf{\Pi}$ *is a cumulative probability matrix if and only if* $\mathbf{\Pi}_{IJ} = 1$ *and*

$$\mathbf{\Pi}_{ij} - \mathbf{\Pi}_{(i-1)j} - \mathbf{\Pi}_{i(j-1)} + \mathbf{\Pi}_{(i-1)(j-1)} \geq 0, \quad 1 \leq i \leq I, 1 \leq j \leq J, \tag{12}$$

where we use the convention that $\mathbf{\Pi}_{0j} = \mathbf{\Pi}_{i0} = 0$.

We continue with some definitions from Nelsen (2007). A review of copulas from a psychometric perspective may be found in the online supplementary material of Foldnes and Grønneberg (2021).

Recall that a copula C is a cumulative distribution function with uniform marginals. That is (Nelsen, 2007, Definition 2.2.2), $C : [0,1]^2 \to [0,1]$ with $C(u,0) = 0 = C(0,v)$, with $C(u,1) = u$ and $C(1,v) = v$ for any $0 \leq u, v \leq 1$, that is also two-increasing. Recall (Nelsen, 2007, Definition 2.1.1) that a two-decreasing function H with the unit square $[0,1]^2$ as its domain is such that for any rectangle $[x_1,x_2] \times [y_1,y_2] \subseteq [0,1]^2$, we have

$$H(x_2,y_2) - H(x_2,y_1) - H(x_1,y_2) + H(x_1,y_1) \geq 0. \tag{13}$$

A copula can be shown to be non-decreasing in each variable and follow the Lipschitz condition

$$|C(u_2,v_2) - C(u_1,v_1)| \leq |u_2 - u_1| + |v_2 - v_1|, \tag{14}$$

for any $(u_1,u_2),(v_1,v_2) \in [0,1]^2$ (Nelsen, 2007, Chapter 2), i.e., copulas are Lipschitz with constant 1.

Let us also recall Sklar's theorem (Sklar, 1959; Nelsen, 2007, Theorem 2.3.3): For any bivariate cumulative distribution F, there exists a copula C such that for any x_1, x_2 we have $F(x_1,x_2) = C(F_1(x_1), F_2(x_2))$ where C is a copula that is unique on the range of F_1, F_2, and therefore unique if F_1, F_2 are continuous. Moreover, if C is a copula and F_1, F_2 are univariate cumulative distribution functions, then $F(x_1,x_2) = C(F_1(x_1), F_2(x_2))$ is a cumulative distribution function with marginals F_1, F_2.

The classical Fréchet–Höffding bound (Nelsen, 2007, Theorem 2.2.3) states that any copula C fulfills $W(u,v) \leq C(u,v) \leq M(u,v)$ for all $u, v \in [0,1]$, where $W(u,v) = \max(u+v-1, 0)$ and $M(u,v) = \min(u,v)$ are themselves copulas.

A quasi-copula (Nelsen, 2007, Definition 6.2.1), is a function $Q : [0,1]^2 \to [0,1]$ with $Q(u,0) = 0 = Q(0,v)$, with $Q(u,1) = u$ and $Q(1,v) = v$ for any $0 \leq u, v \leq 1$, which is non-decreasing in each coordinate, and fulfills the Lipschitz condition of eq. (14). A copula is therefore a quasi-copula.

8. Shuffles of min and the covariance formula

Mikusinski et al. (1992) introduced a class of copulas called *shuffles of min*, henceforth just shuffles. See Nelsen (2007, p. 67) for an overview. After some preliminary definitions, we derive some new results which are used to prove Theorem 1 in Section 11 (p. A15).

Definition 1. Let u and v be vectors in $[0,1]^{K+1}$, where $0 \leq u_1 \leq \cdots u_{K+1} = 1$. Let $s \in \{-1,1\}^K$ be a vector of signs. The function

$$f_{uvs}(x) = \sum_{k=1}^{K} \left\{ v_k + s_k[x - \frac{1}{2}(u_{k+1} + u_k)] + \frac{1}{2}(u_{k+1} - u_k) \right\} 1[u_k \leq x < u_{k+1}] \tag{15}$$

is a *shuffle function* with parameters (u, v, s) if $v_k + (u_{k+1} - u_k) \in v$ for each $i \leq k$.

Remark 2. *(i)* When $s_k = -1$, the summand equals $-x + v_k + u_{k+1}$. At $x \in [u_k, u_{k+1})$, the function starts $v_k + (u_{k+1} - u_k)$ and ends at v_k. When $s_k = 1$, the summand is $x + v_k - u_k$, which starts at v_k and ends at $v_k + (u_{k+1} - u_k)$. *(ii)* From the condition $0 \leq u_1 \leq \cdots u_{k+1} = 1$, we allow the vector u to be non-decreasing instead of strictly increasing. We do this as it makes the results below easier to state. Note that, whenever there are repetitions $u_k = u_{k+1} = \cdots = u_{i+k} < u_{i+k+1}$, the first $(k-1)$ repetitions will be ignored, as $[u_{i+j}, u_{i+j+1}) = \emptyset$ when $0 \leq j \leq k-1$. *(iii)* That $v_k + (u_{k+1} - u_k) \in v$ for each i is equivalent to f_{uvs} being a bijection from $[0,1]$ to $[0,1]$.

We are concerned only with two special kinds of shuffles, the straight shuffle and the flipped shuffle. Let u and v be vectors in $[0,1]^{k+1}$, where $0 \leq u_1 \leq \cdots u_{k+1} = 1$ and $v_k + (u_{k+1} - u_k) \in v$ for each $i \leq k$. Then

$$f_{uv+}(x) = x + \sum_{k=1}^{K} (v_k - u_k) 1[u_k \leq x < u_{k+1}], \tag{16}$$

is a *straight shuffle function* and

$$f_{uv-}(x) = -x + \sum_{k=1}^{K} (v_k + u_{k+1}) 1[u_k \leq x < u_{k+1}], \tag{17}$$

is a *flipped shuffle function*.

Let U be a uniform random variable, f_{uvs} a shuffle function, and define $V = f_{uvs}(U)$. Then V is uniform, hence $P(U \leq u, V \leq v) = C_{uvs}(u,v)$ defines a copula. Define $c_{uvs}(u,v) = 1[v = f_{uvs}(u)]$, the graph of f_{uvs}, and $C_{usv}(u,v) = \int_0^u \int_0^v 1[v = f_{uvs}(u)] du dv$. Then C_{uvs} is the shuffle copula with parameters uvs, and c_{uvs} is its singular copula density.

The shuffle copula has a reasonably tractable formula, as given Carley (2002) and in Genest and Nešlehová (2007, Proposition 2). In our notation, the result is as follows. For completeness, we provide a proof which encompass general shuffle functions.

Proposition 3. *We have*

$$C_{uvs}(u,v) = \sum_{k=1}^{K} \max(\min\{u-u_k, v-v_k, u_{k+1}-u_k, 1[s_k=-1](v-v_k+u-u_{k+1})+1[s_k=1]\}, 0). \tag{18}$$

In particular, the straight shuffle and flipped shuffle have formals

$$C_{uv+}(u,v) = \sum_{k=1}^{K} \max(\min\{u-u_k, v-v_k, u_{k+1}-u_k\}, 0),$$
$$C_{uv-}(u,v) = \sum_{k=1}^{K} \max\{0, -(u_{k+1}-u_k)+\min(u-u_k, u_{k+1}-u_k)+\min(v-v_k, u_{k+1}-u_k)\}.$$

Proof. Write

$$C_{uvs}(u,v) = P(U \le u, f_{uvs}(U) \le v) = \sum_{k=1}^{K} P(u_k \le U \le \min(u_{k+1}, u), f_{uvs}(U) \le v).$$

Let us take a look at the summands, which we will call $g_k(u,v)$. If $s_k = 1$, then $f_{uvs}(U) = U + (v_k - u_k)$, and

$$\begin{aligned} g_k(u,v) &= P(u_k \le U \le \min(u_{k+1}, u), U + (v_k - u_k) \le v), \\ &= P(u_k \le U \le \min(u_{k+1}, u), U \le v - (v_k - u_k)). \end{aligned}$$

Since U is uniform,

$$\begin{aligned} g_k(u,v) &= \max\{\min(u_{k+1}, u, v-(v_k-u_k)) - u_k, 0\}, \\ &= \max\{\min(u_{k+1}-u_k, u-u_k, v-v_k, 0\}. \end{aligned}$$

Supposing that every $s_k = 1$, and taking sums, we find

$$P(U \le u, f_{uv+}(U) \le v) = \sum_{k=1}^{K} \max\{\min(u_{k+1}-u_k, u-u_k, v-v_k, 0\},$$

which is the formula for C_{uv+}.

On the other hand, if $s_k = -1$,

$$\begin{aligned} g_k(u,v) &= P(u_k \le U \le \min(u_{k+1}, u), -U + v_k + u_{k+1} \le v), \\ &= P(u_k \le U \le \min(u_{k+1}, u), v_k + u_{k+1} - v \le U). \end{aligned}$$

Hence

$$g_k(u,v) = \max\{\min(u_{k+1}, u) - \max\{u_k, v_k + u_{k+1} - v)\}, 0\}.$$

We need to show that $g_k(u,v) = h_k(u,v)$, where $h_i(u,v) = \max(\min\{u-u_k, v-v_k, u_{k+1}-u_k, (v - v_k + u - u_{k+1})\}, 0)$. First, consider the case when $u_{k+1} > u$. Then $\min(u_{k+1}, u) = u$ and

$$g_k(u,v) = \max\{\min(u-u_k, u-u_{k+1}+v-v_k)), 0\}.$$

On the other hand, the terms $v - v_k$ dominates $(v - v_k + u - u_{k+1})$ and $u_{k+1} - u_k$ dominates $u - u_k$ in the definition of $h_k(u,v)$, hence $h_k(u,v) = \max\{\min(u-u_k, u-u_{k+1}+v-v_k)), 0\}$ as well. It follows that $g_k(u,v) = h_k(u,v)$. The case when $u_{k+1} < u$ is similar and omitted. □

The following lemma gives formulas for covariances for shuffle copulas. Again, we give the result in the general shuffle case. As far as we know, this result is new.

Lemma 1. *Let C_{uvs} be a shuffle and F_1, F_2 be distribution functions. Recall that $K+1$ is the length of u. The covariance of a distribution with copula C_{uvs} and marginal distributions F_1, F_2 is*

$$\operatorname{Cov}\left(C_{uvs}[F_1, F_2]\right) = \sum_{k=1}^{K} \int_{u_k}^{u_{k+1}} F_1^{-1}(u) F_2^{-1}(f_{uvs}(u)) du - \mu_{F_1}\mu_{F_2}. \tag{19}$$

$$\operatorname{Cov}\left(C_{+vs}[F_1, F_2]\right) = \sum_{k=1}^{K} \int_{u_k}^{u_{k+1}} F_1^{-1}(u) F_2^{-1}(v_k - u_k + u) du, \tag{20}$$

$$\operatorname{Cov}\left(C_{-vs}[F_1, F_2]\right) = \sum_{k=1}^{K} \int_{u_k}^{u_{k+1}} F_1^{-1}(u) F_2^{-1}(v_k + u_{k+1} - u) du, \tag{21}$$

Proof of Lemma 1. Let $f_{uvs}(x)$ be the shuffle function and c_{uvs} the copula density function. From the definition of c_{uvs}, we find that

$$c_{uvs}(F_1(x), F_2(y)) = \begin{cases} 1, & \text{if } F_2(y) = f_{uvs}(F_1(x)), \\ 0, & \text{otherwise.} \end{cases}$$

Let $(X, Y) \sim C_{uvs}[F_1, F_2]$ and calculate the second cross-moment. Then the density of (X, Y) with respect to the one-dimensional measure μ on $A = \{(x, y) \mid F_2(y) = f_{uvs}(F_1(x))\}$ is

$$f(x, y) = \begin{cases} f_1(x) & \text{if } F_2(y) = f_{uvs}(F_1(x)), \\ 0 & \text{otherwise.} \end{cases}$$

It follows from the equation $y = F_2^{-1}(f_{uvs}(F_1(x)))$ that

$$\begin{aligned} EXY &= \int_A f(x, y) xy d\mu, \\ &= \int_A f_1(x) x F_2^{-1}(f_{uv}(F_1(x)) dx. \end{aligned}$$

Define $B = B_1 \cup B_2 \cup \ldots \cup B_K$, where $B_k = [u_k, u_{k+1})$, to obtain

$$\int_A f_1(x) x F_2^{-1}(f_{uv}(F_1(x)) dx = \sum_{k=1}^{K} \int_{F_1^{-1}(B_k)} x F_2^{-1}(v_k - u_k + F_1(x)) f_1(x) dx$$

Now use the substitution $F_1(x) = u$, so that $dx = 1/f(x)du$,

$$EXY = \sum_{k=1}^{K} \int_{u_k}^{u_{k+1}} F_2^{-1}(f_{uvs}(u)) F_1^{-1}(u) du.$$

In particular, for a straight shuffle, $f_{uvs}(F_1(x)) = v_k - u_k + F_1(x)$ on B_k, hence

$$EXY = \sum_{k=1}^{K} \int_{u_k}^{u_{k+1}} F_2^{-1}(v_k - u_k + u) F_1^{-1}(u) du.$$

The formula for a flipped shuffle can be obtained in the same way. □

Now we prove Proposition 1, stating that for any cumulative probability matrix $\mathbf{\Pi}$, the bounds for the latent correlations are $(-1, 1)$ when the marginals F_1 and F_2 are unrestricted.

Proof of Proposition 1. We will only show that the supremum is 1, as the case of the infimum is similar. Let $F = F_1 = F_2$ and use Lemma 1 to split $M_{\mathbf{\Pi}}[F, F]$ into tail moments and a "center moment" $K[F]$.

$$\begin{aligned}\text{Cov } M_{\mathbf{\Pi}}[F, F] &= \sum_{k=1}^{K} \int_{u_k^U}^{u_{k+1}^U} F^{-1}(u)F^{-1}(v_k^U - u_k^U + u)du, \\ &= \int_0^{u_2^U} F^{-1}(u)F^{-1}(u)du + \int_{u_n^U}^{1} F^{-1}(u)F^{-1}(u)du + K[F].\end{aligned}$$

This equation holds since $v_1^U = u_1^U = 0$ and $v_K^U = u_K^U$. To see why this is the case, recall from the definition of u^U (on page 5) that its first element is $\mathbf{\Pi}_{0J} + \mathbf{\Pi}_{10} - \mathbf{\Pi}_{00} = 0$. On the other hand, the first element of v^U is $\mathbf{\Pi}_{I0} + \mathbf{\Pi}_{01} - \mathbf{\Pi}_{00} = 0$ as well. Moreover, the (I, J)th element of both A and B is $\Pi_{(I-1)J} + \Pi_{I(J-1)} - \Pi_{(I-1)(J-1)} = v_K^U = u_K^U$.

Using the substitution $u = F(x)$,

$$\text{Cov } M_{\mathbf{\Pi}}[F, F] = \int_{F^{-1}(0)}^{F^{-1}(u_2^U)} x^2 f(x)dx + \int_{F^{-1}(u_K^U)}^{F^{-1}(1)} x^2 f(x)dx + K[F].$$

Now we construct a sequence of densities f_n (with cdfs F_n) where the tail moments are made arbitrarily large while keeping the "center moment" $K[F_n]$ constant. Define $w_k = F^{-1}(u_k^U)$ and suppose that $-w_2 = w_K$ for simplicity. Define

$$f_n(x) = \begin{cases} u_2^U \cdot 3n^3 x^{-4} & x < -n, \\ (1 - u_2^U - u_K^U) \cdot \frac{1}{w_K - w_2} & x \in [w_2, w_K] \\ u_K^U \cdot 3n^3 x^{-4} & x > n. \end{cases},$$

Provided $n > w_K$, the second moment of $X_n \sim f_n$ is $E[X_n^2] = (u_2^U + u_K^U)n^2 + (1 - u_2^U - u_K^U)\frac{1}{3}\frac{w_K^3 - w_2^3}{w_K - w_2}$. The sum of the tail moment equals $(u_2 + u_K)n^2$, and the mean is 0 since $-w_2 = w_K$. It follows that

$$\text{Cor } M_{\mathbf{\Pi}}[F_n, F_n] = \frac{(u_2^U + u_K^U)n^2 + K[F_n]}{(u_2^U + u_K^U)n^2 + (1 - u_2^U - u_K^U)\frac{1}{3}\frac{w_K^3 - w_2^3}{w_K - w_2}} \to 1.$$

It follows that the maximal correlation is, for any cumulative probability matrix $\mathbf{\Pi}$, equal to $\sup_{F_1,F_2} \text{Cor}(M_{\mathbf{\Pi}}[F_1, F_2]) = 1$. The proof for the minimal correlation is similar and omitted. □

9. Partial identification of latent correlations with compact product set restrictions on the copula

Definition 2. Two bivariate functions C and D *agree* on $\mathcal{S}$ if $C(a, b) = D(a, b)$ at all points $(a, b) \in \mathcal{S}$.

The property of agreeing is preserved under convex combinations, along with the property of being a copula (or quasi-copula). A proof of the following simple convexity lemma can be found at the end of this online appendix.

Lemma 2. *Let D, E be two quasi-copulas. Define the convex combination*

$$C^\lambda(u, v) = \lambda D(u, v) + (1 - \lambda)E(u, v), \quad \lambda \in [0, 1].$$

Then, for every $\lambda \in [0, 1]$, we have that:

(i) If D, E agree with a bivariate function C on $\mathcal{S} \subseteq [0, 1]^2$, then $C^\lambda(u, v)$ agrees with C on $\mathcal{S}$ too.
(ii) C^λ is a quasi-copula.
(iii) If D, E are proper copulas, then C^λ is a proper copula as well.

Let $\mathcal{S}$ be a subset of $[0,1]^2$ and C be a copula, and recall the notation $x^+ := \max(x, 0)$. Define the quasi-copulas

$$M_{\mathcal{S};C}(u,v) = \min\left\{u, v, \min_{(a,b)\in\mathcal{S}}[C(a,b) + (u-a)^+ + (v-b)^+]\right\}, \tag{22}$$

$$W_{\mathcal{S};C}(u,v) = \max\left\{0, u+v-1, \max_{(a,b)\in\mathcal{S}}[C(a,b) - (a-u)^+ - (b-v)^+]\right\}. \tag{23}$$

That these functions are quasi-copulas follows from Proposition 1 by Tankov (2011). The following reformulation of Theorem 2 of Bernard et al. (2013) will be used repeatedly in the sequel.

Proposition 4. *Let $\mathcal{S} = A \times B$ for two compact sets $A, B \subseteq [0,1]$ and let C be a copula. Then*

(i) $M_{\mathcal{S};C}$ and $W_{\mathcal{S};C}$ agree with C on $\mathcal{S}$,
(ii) $M_{\mathcal{S};C}$ and $W_{\mathcal{S};C}$ are copulas,
(iii) If D agrees with C on S, then

$$W_{\mathcal{S};C}(u,v) \leq D(u,v) \leq M_{\mathcal{S};C}(u,v), \quad (u,v)\in[0,1]^2. \tag{24}$$

Proof. The proposition is a consequence of Proposition 1 in Tankov (2011), except the conclusion that $M_{\mathcal{S};C}, W_{\mathcal{S};C}$ are proper copulas, not just quasi-copulas. This in turn follows from Theorem 2 Bernard et al. (2013), where the assumptions are (i) that $\mathcal{S}$ is compact since both A and B are compact, and (ii) whenever $(a_1, b_1) \in \mathcal{S}$ and $(a_2, b_2) \in \mathcal{S}$, then $(a_1, b_2) \in \mathcal{S}$ and $(a_2, b_1) \in \mathcal{S}$. But by Lemma 7, (ii) is equivalent to $\mathcal{S} = A \times B$, and we are done. □

We can connect Proposition 4 to the convexity Lemma 2 to find a large class of copulas agreeing with C on $\mathcal{S}$. For the upper bound $M_{\mathcal{S};C}$ and lower bound $W_{\mathcal{S};C}$ in the proposition above, and any $\lambda \in [0,1]$, define the convex combination

$$C^{\lambda}_{\mathcal{S};C}(u,v) = \lambda M_{\mathcal{S};C}(u,v) + (1-\lambda)W_{\mathcal{S};C}(u,v). \tag{25}$$

Corollary 3. *Let $\mathcal{S} = A \times B$ for two compact sets $A, B \subseteq [0,1]$ and let C be a copula. For any $\lambda \in [0,1]$, the convex combination $C^{\lambda}_{\mathcal{S};C}$ is a copula that agrees with C on $\mathcal{S}$.*

Let $\rho(F)$ denote the Pearson correlation of a random variable with bivariate distribution function F. Let F_1, F_2 be cumulative distribution functions, C be a copula, and $\mathcal{S}$ be a subset of $[0,1]^2$. Define

$$\rho(\mathcal{S}; C, F_1, F_2) = \{\rho(F) \mid F \text{ has marginals } F_1, F_2 \text{ and copula } C_F \text{ that agrees with } C \text{ on } \mathcal{S}\}.$$

Our current task is to find a natural and computable way to represent $\rho(\mathcal{S}; C, F_1, F_2)$ when $\mathcal{S} = A \times B$ for compact A, B.

When Q is a quasi-copula, define the function $Q[F_1, F_2]$ on the domain of F_1 and F_2 by $Q[F_1, F_2](x,y) = Q(F_1(x), F_2(x))$. If $Q = C$ is a copula, then $C[F_1, F_2]$ is a bivariate distribution function. If F is a bivariate distribution with marginals F_1, F_2 and copula C_F, then $F = C_F[F_1, F_2])$ by Sklar's theorem.

Theorem 3. *Let F_1, F_2 be continuous and strictly increasing distribution functions, C a copula, and $\mathcal{S} = A \times B$ for two compact sets $A, B \subseteq [0,1]$. Then*

$$\rho(\mathcal{S}; C, F_1, F_2) = [\rho(W_{\mathcal{S},C}[F_1, F_2]), \rho(M_{\mathcal{S},C}[F_1, F_2])].$$

Proof. From Proposition 4 and the remarks above, $W_{\mathcal{S};C}[F_1, F_2]$ and $M_{\mathcal{S};C}[F_1, F_2]$ are bivariate distribution functions with marginals F_1, F_2 whose copulas agree with C on $\mathcal{S}$.

Provided the marginals F_1, F_2 are fixed, we see from the Höffding correlation formula (11) (on p. 8) that the correlation is a monotone function of copulas in the sense that $\rho(C_1[F_1, F_2]) \leq \rho(C_2[F_1, F_2])$ whenever $C_1(u, v) \leq C_2(u, v)$ for all $0 \leq u, v \leq 1$. Since $M_{\mathcal{S};C}[F_1, F_2]$ is the upper bound of copulas agreeing with C and $W_{\mathcal{S};C}[F_1, F_2]$ is the lower bound of copulas agreeing with C, we find that

$$\begin{aligned} \min \rho(\mathcal{S}; C, F_1, F_2) &= \rho(W_{\mathcal{S};C}[F_1, F_2]), \\ \max \rho(\mathcal{S}; C, F_1, F_2) &= \rho(M_{\mathcal{S};C}[F_1, F_2]). \end{aligned}$$

From Corollary 3, we know that the convex combination $C^{\lambda}_{\mathcal{S};C}$ agrees with C on $\mathcal{S}$. Thus $f(\lambda) = \rho(C^{\lambda}_{\mathcal{S};C}[F_1, F_2]) \in \rho(\mathcal{S}; C, F_1, F_2)$ for all λ as well. But it follows from the Höffding correlation formula that

$$f(\lambda) = \lambda\rho(M_{\mathcal{S};C[F_1,F_2]}) + (1 - \lambda)\rho(W_{\mathcal{S};C}[F_1, F_2]).$$

Hence any correlation between $\rho(M_{\mathcal{S};C}[F_1, F_2])$ and $\rho(W_{\mathcal{S};C}[F_1, F_2])$ can be attained. It follows that $\rho(\mathcal{S}; C, F_1, F_2) = [\rho(W_{\mathcal{S},C}[F_1, F_2]), \rho(M_{\mathcal{S},C}[F_1, F_2])]$, and we are done. □

9.1. **Alternative formulas for Theorem 1.** We show that the general framework of Theorem 3 fits in with the restrictions we know in the ordinal case, as considered in Section 2 .

Recall eq. (2) (p. 4), which gives us the restrictions

$$\boldsymbol{\Pi}_{ij} = C(\boldsymbol{\Pi}_{iJ}, \boldsymbol{\Pi}_{Ij}), \quad (i = 1, \ldots, I - 1,\ j = 1, \ldots, J - 1) \tag{26}$$

on C, the copula of Z. In other words, we know the value of $C(u, v)$ for (u, v) in

$$\mathcal{S} = \{\boldsymbol{\Pi}_{iJ} : 1 \leq i \leq I - 1\} \times \{\boldsymbol{\Pi}_{Ij} : 1 \leq j \leq J - 1\}, \tag{27}$$

where "×" is the Cartesian product. This set is a compact product set, and Theorem 3 directly applies.

Here we have removed the conditions corresponding to $i = I$ and $j = J$ from equation (26), as they are automatically satisfied by any copula. For instance, $\boldsymbol{\Pi}_{Ij} = C(1, \boldsymbol{\Pi}_{Ij}) = C(\boldsymbol{\Pi}_{IJ}, \boldsymbol{\Pi}_{Ij})$ trivially, since all copulas have uniform marginals. The set $\mathcal{S}$ of eq. (27) therefore identifies all that is known of the copula C based on the known distribution $\boldsymbol{\Pi}$.

When directly using Theorem 3 with $\mathcal{S}$ of eq. (27), the limit copulas from eq. (22) and eq. (23) specialize to

$$W_{\boldsymbol{\Pi}}(u, v) = \max\left(0, u + v - 1, \max_{1 \leq i < I-1} \max_{1 \leq j < J-1} \left[\boldsymbol{\Pi}_{ij} - (\boldsymbol{\Pi}_{iJ} - u)^+ - (\boldsymbol{\Pi}_{Ij} - v)^+\right]\right), \tag{28}$$

$$M_{\boldsymbol{\Pi}}(u, v) = \min\left(u, v, \min_{1 \leq i < I-1} \min_{1 \leq j < J-1} \left[\boldsymbol{\Pi}_{ij} + (u - \boldsymbol{\Pi}_{iJ})^+ + (v - \boldsymbol{\Pi}_{Ij})^+\right]\right), \tag{29}$$

which are improved versions of the Fréchet–Höffding copulas that fulfill the restrictions in eq. (26). That is, they are copulas compatible with $\boldsymbol{\Pi}$, which fulfill

$$W_{\boldsymbol{\Pi}}(u, v) \leq C(u, v) \leq M_{\boldsymbol{\Pi}}(u, v), \quad 0 \leq u, v \leq 1. \tag{30}$$

Due to eq. (30), the copulas $W_{\boldsymbol{\Pi}}$ and $M_{\boldsymbol{\Pi}}$ are extremes in the space copulas that are compatible with $\boldsymbol{\Pi}$.

Now we derive some simplifications for the limit copulas $W_{\boldsymbol{\Pi}}$ and $M_{\boldsymbol{\Pi}}$ from equations (28) and (29) respectively. These simplified formulas are derived in a similar manner to the derivations in the proof of Lemma 2 in Grønneberg et al. (2020). They will be used in proving Corollary 1 (p.7), Corollary 2 (p.8), and results for when the copula is known to be symmetric. However, they will not be used to prove Theorem 1 in the upcoming Section 11, as this result will apply the shuffle of min framework developed in Section 8, which will reach the simple formulas for the limit correlations that is given in the statement of Theorem 1.

In $W_{\mathbf{\Pi}}$, the inner maximum runs over $(I-1)(J-1)$ variables, but can be reduced to just 4. Define $i(u)$ to be the largest index i satisfying $\mathbf{\Pi}_{iJ} < u$ and define $\mathcal{I}(u) = \{i(u), i(u)+1\}\backslash\{0, I\}$. Likewise, define $j(v)$ to be the largest index i satisfying $\mathbf{\Pi}_{Ij} < v$ and define $\mathcal{J}(v) = \{j(v), j(v)+1\}\backslash\{0, J\}$. Then the limit copulas can be written as

$$W_{\mathbf{\Pi}}(u,v) = \max\left(0, u+v-1, \max_{i\in\mathcal{I}(u)}\max_{j\in\mathcal{J}(v)}\left[\mathbf{\Pi}_{ij} - (\mathbf{\Pi}_{iJ}-u)^{+} - (\mathbf{\Pi}_{Ij}-v)^{+}\right]\right), \tag{31}$$

$$M_{\mathbf{\Pi}}(u,v) = \min\left(u, v, \min_{i\in\mathcal{I}(u)}\min_{j\in\mathcal{J}(v)}\left[\mathbf{\Pi}_{ij} + (u-\mathbf{\Pi}_{iJ})^{+} + (v-\mathbf{\Pi}_{Ij})^{+}\right]\right). \tag{32}$$

The proof of the simplification is now given, but only for W, as the as proof for M is the same. Consider $W(u,v)$, whose inner maximum is

$$\max_{1\leq i<I-1}\max_{1\leq j<J-1}\left(\mathbf{\Pi}_{ij} - (\mathbf{\Pi}_{iJ}-u)^{+} - (\mathbf{\Pi}_{Ij}-v)^{+}\right) =$$
$$\max_{1\leq i<I-1}\left(\max_{1\leq j<J-1}[\mathbf{\Pi}_{ij} - (\mathbf{\Pi}_{Ij}-v)^{+}] - (\mathbf{\Pi}_{iJ}-u)^{+}\right).$$

Here

$$\max_{1\leq j<J} g_{iv}(j) = \max_{1\leq j<J}[\mathbf{\Pi}_{ij} - (\mathbf{\Pi}_{jI}-v)^{+}] = \max_{1\leq j<J}\begin{cases}\mathbf{\Pi}_{ij} & v\geq \mathbf{\Pi}_{Ij},\\ \mathbf{\Pi}_{ij} - \mathbf{\Pi}_{Ij} + v & v<\mathbf{\Pi}_{Ij}.\end{cases}$$

is a function of i and v only. Since $\mathbf{\Pi}_{ij}$ increases in i, j, the function $g_{iv}(j)$ increases in j until $v \geq \mathbf{\Pi}_{Ij}$. On the other hand, $\mathbf{\Pi}_{ij} - \mathbf{\Pi}_{Ij}$ is non-increasing in j, see Lemma 3 in Grønneberg et al. (2020) for a proof. This leaves $j(v)$ and $j(v)+1$ as the potential maximizers of $g_{iv}(j)$. The same analysis can be done for i, leaving us with

$$\max_{i\in\{i(u),i(u)+1\}}\max_{j\in\{j(v),j(v)+1\}}\left(\mathbf{\Pi}_{ij} - (\mathbf{\Pi}_{iJ}-u)^{+} - (\mathbf{\Pi}_{Ij}-v)^{+}\right). \tag{33}$$

10. Known marginals with symmetric copulas

Consider the case where we have substantive knowledge of the distribution of Z in the form that the copula of Z is a symmetric copula, in addition to knowing the marginals of Z. A copula C is symmetric if it fulfills $C(u,v) = C(v,u)$ for all $(u,v) \in [0,1]^2$ (Nelsen, 1993, 2007). Many well-known parametric bivariate copulas are symmetric (Li & Genton, 2013, Figure 1), including the normal copula.

The polychoric correlation estimator (Olsson, 1979) is derived under the assumption that Z is bivariate normal. In other words, each marginal is standard normal, and the copula of Z is the normal copula. In this section we assume that the psychometrician knows the marginal distribution of Z and that the copula is symmetric, but does not know enough about the distribution to conclude with bivariate normality or some other parametric class of distributions whose parameters are identifiable from $\mathbf{\Pi}$.

In comparison to the partial identification sets of the previous section, the additional assumption of a symmetric copula leads to smaller partial identification sets for the latent correlation, yet as we will see, not by much. The normal copula also fulfill the assumption known as radial symmetry (Li & Genton, 2013), and is an elliptical copula. The properties of symmetry, radial symmetry and ellipticalness are examples of classes of copulas, and one can naturally ask the question how partial identification sets are influenced by different types of assumptions on the copula and/or marginals. Future research may lead to a calculus for partial identification analysis based on the specification of distributional classes that are seen as realistic, which could lead to small and informative partial identification sets.

Just as there are cumulative probability matrices $\mathbf{\Pi}$ that are not compatible with latent bivariate normality, there are matrices not compatible with any symmetric copula. For instance, the matrix

$$\mathbf{\Pi} = \begin{bmatrix} 0.01 & 0.10 & 0.10 \\ 0.05 & 0.25 & 0.40 \\ 0.10 & 0.40 & 1.00 \end{bmatrix}$$

is not compatible with any symmetric copula: From eq. (26) (p. A7), we know that any copula $C(u, v)$ compatible with $\mathbf{\Pi}$ will satisfy $C(0.40, 0.10) = 0.10$ and $C(0.10, 0.40) = 0.05$, but this violates the symmetry constraint $C(u, v) = C(v, u)$. We will assume that $\mathbf{\Pi}$ is compatible with some symmetric C from now on. Similar to the normality assumption, the assumption of a symmetric latent copula has testable implications. The problem of testing this assumption is considered outside the scope of the present paper.

Theorem 4 below solves the partial identification problem considered in this section. The solution is given in terms of a class of matrices which we call symmetric extensions of cumulative probability matrices. In the definition of this class, we use the notions of a submatrix and an ordered set-theoretic union, which we now recall. By a submatrix of a matrix A, we mean a matrix formed from A by selecting a subset of its rows and columns. By the ordered set-theoretic union $u \sqcup v$ of two vectors u, v, we mean the vector resulting in first computing the set-theoretic union of the elements in u, v, and then sorting the elements of this union. Recall that the set-theoretic union discards duplicates. For instance, $(0.1, 0.3, 0.9, 1) \sqcup (0.2, 0.5, 0.9, 1) = (0.1, 0.2, 0.3, 0.5, 0.9, 1)$.

Definition 3. Let $\mathbf{\Pi}$ be a cumulative probability matrix. A matrix $\mathbf{\Pi}^S$ is a *symmetric extension* of $\mathbf{\Pi}$ if

(1) The matrix $\mathbf{\Pi}^S$ is symmetric, and its final row and column both equals $\mathbf{\Pi}_{I\cdot} \sqcup \mathbf{\Pi}_{\cdot J}$ where $\mathbf{\Pi}_{I\cdot}$ is the final row of $\mathbf{\Pi}$ and $\mathbf{\Pi}_{\cdot J}$ is the final column of $\mathbf{\Pi}$.
(2) $\mathbf{\Pi}$ is a submatrix of $\mathbf{\Pi}^S$.
(3) $\mathbf{\Pi}^S$ is a cumulative probability matrix.

Requirement (1) determines the dimensions of $\mathbf{\Pi}^S$, and so all symmetric extensions of $\mathbf{\Pi}$ have the same dimensionality, and are always square matrices. A general description of how to identify all symmetric extensions of a given $\mathbf{\Pi}$, as well as a concrete numerical example can be found at the start of Section 10.1.

Remark 3. The above definition defines a class of matrices that describe the set of ordinal distributions that can be generated by an latent symmetric copula in a minimal way. Requirement (3) means that the matrix induces a valid probability distribution. Requirement (2) incorporates the restrictions on C we know from eq. (26) (p. A7). Requirement (1) ensures that all information derivable from the symmetry assumption is taken into account. Since this encoding is minimal, in the sense that none of these demands can be weakened without losing information on C known to hold from the original matrix $\mathbf{\Pi}$ or the symmetry of C, symmetric extensions could therefore also be named minimal symmetric extensions.

Now we state the main result of this section.

Theorem 4. *Let $\mathcal{F}_S$ be the set of distributions with symmetric copulas and continuous and strictly increasing marginals F_1, F_2, and let $\mathcal{S}$ be the set of symmetric extensions of a cumulative probability matrix $\mathbf{\Pi}$. Further suppose $\mathbf{\Pi}$ can be generated by a symmetric copula. Then*

$$\rho_{\mathbf{\Pi}}(\mathcal{F}_S) = [\min_{A \in \mathcal{S}} \rho(W_A; F_1, F_2), \max_{A \in \mathcal{S}} \rho(M_A; F_1, F_2)], \tag{34}$$

where W_A and M_A are the improved versions of the Fréchet–Höffding bounds from eq. (28) and eq. (29) (p. A7).

Proof. See Appendix 10.1, page A11 □

We may formulate the the lower limit correlation as an optimization problem:

$$\begin{aligned} \text{minimize} \quad & \rho(W_{\mathbf{\Pi}^S}; F_1, F_2), \\ \text{subject to} \quad & \mathbf{\Pi}^S \text{ is a symmetric extension of } \mathbf{\Pi}. \end{aligned}$$

The upper bound can be found by replacing "minimize" with "maximize", and W with M. As shown in Section 10.1, the constraints in the optimization problem are linear. Since ρ is differentiable in $\mathbf{\Pi}^S$, the problem therefore can be solved using constrained linear optimization routines, such as the function `stats` :: `constrOptim` included in `R`. The `polyiden` function in the `R` package `polyiden` accompanying this paper can calculate these bounds. However, the current implementation requires an unreasonable amount of computation time for most cases of practical interest.

We have therefore identified an approximation to the problem using a minor modification of the copula bounds for the asymmetric case. The resulting approximation is numerically quick to compute. Assume $C(u,v)$ is symmetric and $C(u,v) \leq D(u,v)$ for all $0 \leq u,v \leq 1$ for some potentially asymmetric copula D. Then $C(v,u) = C(u,v) \leq D(u,v)$ as well, hence $C(u,v) \leq \min[D(u,v), D(v,u)]$. Moreover, $\min[D(u,v), D(v,u)]$ is symmetric. Similarly, if $D(u,v) \leq C(u,v)$ for all $0 \leq u,v \leq 1$, we find that $\max[D(u,v), D(v,u)] \leq C(u,v)$, with $\max[D(u,v), D(v,u)]$ symmetric. Recalling the limit copulas in the asymmetric case, this suggest that the symmetric limit copulas are

$$W^S_{\mathbf{\Pi}}(u,v) = \max[W_{\mathbf{\Pi}}(u,v), W_{\mathbf{\Pi}}(v,u)], \quad M^S_{\mathbf{\Pi}}(u,v) = \min[M_{\mathbf{\Pi}}(u,v), M_{\mathbf{\Pi}}(v,u)], \tag{35}$$

where $W_{\mathbf{\Pi}}$ and $M_{\mathbf{\Pi}}$ are the copulas defined in the equations (28) and (29). However, this fails to be the case, as these bounds typically fail to be copulas. Theorem 6.2.5 in Nelsen (2007) implies that maxima and minima of copulas are quasi-copulas (see Section 7, p. A1, for the definition of quasi-copulas), but need not be proper copulas. In the present case, $W^S_{\mathbf{\Pi}}$ and $M^S_{\mathbf{\Pi}}$ are not copulas but almost always proper quasi-copulas, as can be verified numerically. Therefore the bounds generated by $W^S_{\mathbf{\Pi}}$ and $M^S_{\mathbf{\Pi}}$ cannot be used to calculate partial identification sets. All they can do is to provide bounds.

Using the same reasoning as when proving Theorem 1, we find a superset of the identification set for the latent correlation under the assumption of symmetric copulas. This superset is quick to compute, and may be calculated using the `polyiden` function in the `polyiden` package.

Proposition 5. *Let $\mathcal{F}_S$ be the set of distributions with symmetric copulas and continuous and strictly increasing marginals F_1, F_2. Then $\rho_{\mathbf{\Pi}}(\mathcal{F}_S) \subseteq [\rho(W^S_{\mathbf{\Pi}}; F_1, F_2), \rho(M^S_{\mathbf{\Pi}}; F_1, F_2)]$.*

Proof. By part (i) and (ii) of Lemma 2 on p. A5, the convex combination $C^{\lambda}_{\mathbf{\Pi}}(u,v) = \lambda W^S_{\mathbf{\Pi}}(u,v) + (1-\lambda) M^S_{\mathbf{\Pi}}(u,v)$ is a quasi-copula compatible with $\mathbf{\Pi}$ for any $\lambda \in [0,1]$. Since symmetry is preserved under convex combinations, $C_{\mathbf{\Pi}}(u,v;\lambda)$ is also a symmetric quasi-copula. The rest of the proof is similar to that of Theorem 3 on p. A6 and is omitted. □

The difference between the true identification set and the approximation of Proposition 5 tends, in our numerical experiments, to be insignificant. For example, taking the 2×2 matrix in equation (36) in the numerical example provided in Example 2 (Section 10.1, p. A11), the partial identification set is $\rho_{\mathbf{\Pi}}(\mathcal{F}_S) = [-0.8376, 0.8786]$, while the approximation from Proposition 5 is $[\rho(W^S_{\mathbf{\Pi}}; F_1, F_2), \rho(M^S_{\mathbf{\Pi}}; F_1, F_2)] = [-0.8541, 0.8786]$. Without a symmetry assumption, we get from Theorem 1 that $\rho_{\mathbf{\Pi}}(\mathcal{F}) = [-0.9137, 0.9137]$.

The similarity between the approximation and the exact computation of the identification sets hold, in our experience, for larger values of I and J too. But this conclusion is based on non-extensive experimentation due to the long computation time for the exact solution.

Unfortunately, adding the symmetry assumption makes little difference for the size of the identification set for ρ. Using the approximation to the symmetric identification sets, Figure 5 shows what happens to the partial identification sets when using the computationally feasible approximate partial identification set for symmetrical copulas (red circles) and arbitrary copulas (black squares) when the marginals are normal, and the remaining setup of the numerical illustration is as in Example 1 (p. 5). The information gained from assuming symmetry appears negligible.

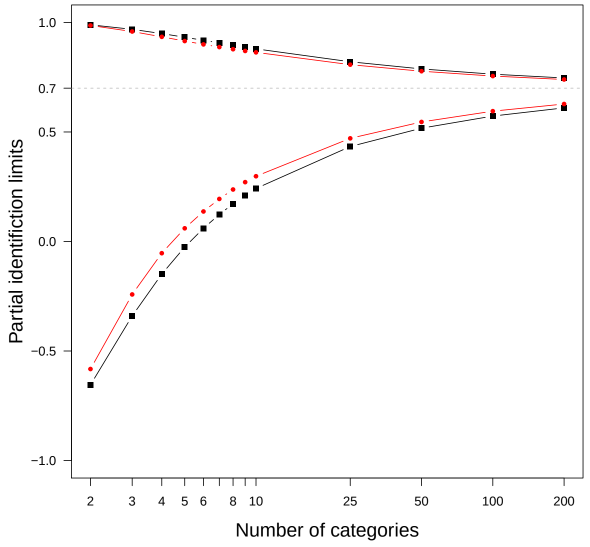

FIGURE 5

Upper and lower limits for the partial identification set with normal marginals, and with (red circles; quasi-copulas) and without (black squares) symmetry.

10.1. **Proofs and technical details for Section 10, and the proof of Theorem 4.** We first consider how to identify symmetric extensions of a given $\mathbf{\Pi}$ and give a numerical example showing how to compute symmetric extensions in a simple case. We then give a series of lemmas, and use them to prove Theorem 4.

It follows from Proposition 2 that a matrix $\mathbf{\Pi}^S$ is a symmetric extension of $\mathbf{\Pi}$ if and only if it satisfies conditions (1) and (2) of Definition 3 and the constraints of equation (12).

Example 2. For an example, let us consider the cumulative probability matrix

$$\mathbf{\Pi} = \begin{bmatrix} 0.4 & 0.8 \\ 0.5 & 1 \end{bmatrix}. \tag{36}$$

A symmetric extension $\mathbf{\Pi}^S$ will have final row and final column equal to $(0.5,1) \sqcup (0.8,1) = (0.5,0.8,1)$, be symmetric, and have $\mathbf{\Pi}$ as a submatrix. Hence there are some x_1, x_2 so that

$$\mathbf{\Pi}^S(C) = \begin{bmatrix} x_1 & 0.4 & 0.5 \\ 0.4 & x_2 & 0.8 \\ 0.5 & 0.8 & 1 \end{bmatrix}. \tag{37}$$

Recall that a submatrix of a matrix A as a matrix formed from A by selecting a subset of its rows and columns. By selecting the first and third column and the second and third row of $\mathbf{\Pi}^S(C)$., we see that $\mathbf{\Pi}$ is a submatrix of $\mathbf{\Pi}^S(C)$.

The constraints of eq. (12) (p. A1) lead to

$$0 \leq x_1 \leq 0.4, \quad 0.6 \leq x_2 \leq 0.7 \quad x_1 + x_2 \geq 0.8.$$

Letting x_1, x_2 vary according to these restrictions means eq. (37) provides a complete description of the space of symmetric extensions $\mathbf{\Pi}^S$ of $\mathbf{\Pi}$ in eq. (36). The same argument identifies the symmetric extension of any matrix, which again will amount to sets of linear inequalities as above. A general algorithm for calculating the symmetric extension of a given matrix is provided within the `polyiden` function in the `R` package `polyiden` accompanying this paper. □

The following lemma will be useful. Point (ii) of the following Lemma is crucial, as it shows that symmetry of $\mathbf{\Pi}$ implies that $M_{\mathbf{\Pi}}, W_{\mathbf{\Pi}}$ are symmetric copulas, connecting two separate concepts of symmetry. This correspondence between a property of cumulative probability matrices and copulas does not need to be true with other types of structural assumptions, which would in general complicate partial identification analyses. This crucial property is used in eq. (40) in the proof of the upcoming Lemma 4.

Lemma 3. *Let $\mathbf{\Pi}$ be a cumulative probability matrix and $\mathbf{\Pi}^T$ its transpose. Then*

(i) $W_{\mathbf{\Pi}^T}(u,v) = W_{\mathbf{\Pi}}(v,u)$ and $M_{\mathbf{\Pi}^T}(u,v) = M_{\mathbf{\Pi}}(v,u)$.

(ii) If $\mathbf{\Pi}$ is symmetric, then $W_{\mathbf{\Pi}}(u,v) = W_{\mathbf{\Pi}}(v,u)$ and $M_{\mathbf{\Pi}}(u,v) = M_{\mathbf{\Pi}}(v,u)$, that is, $M_{\mathbf{\Pi}}$ and $V_{\mathbf{\Pi}}$ are symmetric copulas.

(iii) If the cumulative probability matrix $\mathbf{\Pi}'$ is a submatrix of $\mathbf{\Pi}$ and C is compatible with $\mathbf{\Pi}$, then C then is compatible with $\mathbf{\Pi}'$ too.

Proof. We prove the results for M only; W is similar. Point (ii) follows from (i), as $M_{\mathbf{\Pi}}(u,v) = M_{\mathbf{\Pi}^T}(v,u) = M_{\mathbf{\Pi}}(u,v)$. That (i) holds follows directly from the expression for $M_{\mathbf{\Pi}}$: Assuming $\mathbf{\Pi}$ is an $I \times J$ matrix, we find that

$$\begin{aligned} M_{\mathbf{\Pi}^T}(u,v) &= \min(u, v, \min_{1\leq i\leq I-1} \min_{1\leq j\leq J-1} [\mathbf{\Pi}^T_{ji} + (u - \mathbf{\Pi}^T_{jI})^+ + (v - \mathbf{\Pi}^T_{Ji})^+], \\ &= \min(u, v, \min_{1\leq i\leq I-1} \min_{1\leq j\leq J-1} [\mathbf{\Pi}_{ij} + (u - \mathbf{\Pi}_{Ij})^+ + (v - \mathbf{\Pi}_{iJ})^+], \\ &= \min(u, v, \min_{1\leq i\leq I-1} \min_{1\leq j\leq J-1} [\mathbf{\Pi}_{ij} + (v - \mathbf{\Pi}_{iJ})^+ + (u - \mathbf{\Pi}_{Ij})^+], \\ &= M_{\mathbf{\Pi}}(v,u). \end{aligned}$$

(iii) Assume that $\mathbf{\Pi}'$ is a $I' \times J'$ cumulative probability matrix and a submatrix of the $I \times J$ matrix $\mathbf{\Pi}$. Let $(l_1, l_2, \ldots, I)$ be the row indices and $(k_1, k_2, \ldots, J)$ the column indices of $\mathbf{\Pi}'$, so that $\mathbf{\Pi}'_{ij} = \mathbf{\Pi}_{l_k k_l}$. Then $C(\mathbf{\Pi}'_{iJ'}, \mathbf{\Pi}'_{I'j}) = C(\mathbf{\Pi}_{l_i J}, \mathbf{\Pi}_{I k_j})$. Since C is compatible with $\mathbf{\Pi}$, we have that $C(\mathbf{\Pi}_{l_i J}, \mathbf{\Pi}_{I k_j}) = \mathbf{\Pi}_{l_i k_j}$ too. But since $\mathbf{\Pi}_{l_i k_j} = \mathbf{\Pi}'_{ij}$ we have that $C(\mathbf{\Pi}'_{iJ'}, \mathbf{\Pi}'_{I'j}) = \mathbf{\Pi}'_{ij}$, which shows that C is compatible with $\mathbf{\Pi}'$. □

Let C be a copula and $\mathfrak{u}$, $\mathfrak{v}$ be increasing vectors with positive elements and whose final element is 1. Define $\mathbf{\Pi}(C;\mathfrak{u},\mathfrak{v})$ as the matrix with elements $\mathbf{\Pi}(C;\mathfrak{u},\mathfrak{v})_{ij} = C(\mathfrak{u}_i,\mathfrak{v}_j)$. Then $\mathbf{\Pi}(C;\mathfrak{u},\mathfrak{v})$ is seen to be a cumulative probability matrix. Notice that thanks to eq. (26) and the product structure of the $\mathcal{S}$ restrictions in eq. (27) (p. A7), we have that for any copula C that is compatible with $\mathbf{\Pi}$ the equation

$$\mathbf{\Pi} = \mathbf{\Pi}(C;\mathbf{\Pi}_{\cdot J},\mathbf{\Pi}_{I\cdot})$$

holds, where $\mathbf{\Pi}_{\cdot J}$ has elements $\mathbf{\Pi}_{iJ}$ for $1 \leq i \leq I$ and $\mathbf{\Pi}_{I\cdot}$ has elements $\mathbf{\Pi}_{Ij}$ for $1 \leq j \leq J$.

Similarly, for a given C that is compatible with $\mathbf{\Pi}$, we define its induced symmetric extension of $\mathbf{\Pi}$ as

$$\mathbf{\Pi}^S(C) := \mathbf{\Pi}(C;\mathbf{\Pi}_{I\cdot}\sqcup\mathbf{\Pi}_{\cdot J},\mathbf{\Pi}_{I\cdot}\sqcup\mathbf{\Pi}_{\cdot J}) \tag{38}$$

which will shortly be shown to be a symmetric cumulative probability matrix.

Lemma 4. *Let $\mathbf{\Pi}^S(C)$ be as in eq. (38), and let F_1, F_2 be continuous and strictly increasing univariate cumulative distribution functions. Also let $\mathcal{C} = \mathcal{C}(\mathbf{\Pi})$ be the set of all symmetric copulas which are compatible with $\mathbf{\Pi}$. Then*

$$\begin{aligned} \inf_{C\in\mathcal{C}} \rho(C;F_1,F_2) &= \inf_{C\in\mathcal{C}} \rho(W_{\mathbf{\Pi}^S(C)};F_1,F_2), \\ \sup_{C\in\mathcal{C}} \rho(C;F_1,F_2) &= \sup_{C\in\mathcal{C}} \rho(M_{\mathbf{\Pi}^S(C)};F_1,F_2). \end{aligned} \tag{39}$$

Proof. We consider the lower limit, $\inf_{C\in\mathcal{C}} \rho(W_{\mathbf{\Pi}^S(C)};F_1,F_2)$; the upper limit follows in the same way.

Let C be a symmetric copula compatible with $\mathbf{\Pi}$. Use Theorem 1 with the cumulative probability matrix $\mathbf{\Pi}^S(C)$ and the distribution set $\tilde{\mathcal{F}}$, where $\tilde{\mathcal{F}}$ is the class of distributions with marginals F_1, F_2, but without the symmetry assumption on the copula. This shows that $\rho(W_{\mathbf{\Pi}^S(C)};F_1,F_2)$ is the smallest correlation attainable for any copula (symmetric or not) compatible with $\mathbf{\Pi}^S(C)$. Since C is compatible with $\mathbf{\Pi}^S(C)$, it follows that $\rho(C;F_1,F_2) \geq \rho(W_{\mathbf{\Pi}^S(C)};F_1,F_2)$. Taking infimums on both sides, we obtain

$$\inf_{C\in\mathcal{C}} \rho(C;F_1,F_2) \geq \inf_{C\in\mathcal{C}} \rho(W_{\mathbf{\Pi}^S(C)};F_1,F_2).$$

Since $\mathbf{\Pi}$ is a submatrix of $\mathbf{\Pi}^S(C)$ whenever C is compatible with $\mathbf{\Pi}$, the copula $W_{\mathbf{\Pi}^S(C)}$, which is compatible with $\mathbf{\Pi}^S(C)$, is also compatible with $\mathbf{\Pi}$. Now $W_{\mathbf{\Pi}^S(C)}$ is a copula compatible with $\mathbf{\Pi}^S(C)$. Since $\mathbf{\Pi}$ is a submatrix of $\mathbf{\Pi}^S(C)$, the copula $W_{\mathbf{\Pi}^S(C)}$ is also compatible with $\mathbf{\Pi}$ by Lemma 3 (iii).

Since $\mathbf{\Pi}^S(C)$ symmetric, the copula $W_{\mathbf{\Pi}^S(C)}$ is a symmetric copula by Lemma 3 (ii) (p. A12). Therefore,

$$W_{\mathbf{\Pi}^S(C)} \in \mathcal{C}, \tag{40}$$

and it follows that $\inf_{C\in\mathcal{C}} \rho(C;F_1,F_2) \leq \rho(W_{\mathbf{\Pi}^S(C)};F_1,F_2)$. Now take the infimum on the right-hand side to obtain

$$\inf_{C\in\mathcal{C}} \rho(C;F_1,F_2) \leq \inf_{C\in\mathcal{C}} \rho(W_{\mathbf{\Pi}^S(C)};F_1,F_2).$$

As both $\inf_{C\in\mathcal{C}} \rho(C;F_1,F_2) \leq \inf_{C\in\mathcal{C}} \rho(W_{\mathbf{\Pi}^S(C)};F_1,F_2)$ and $\rho_l \geq \inf_{C\in\mathcal{C}} \rho(W_{\mathbf{\Pi}^S(C)};F_1,F_2)$, we conclude $\inf_{C\in\mathcal{C}} \rho(C;F_1,F_2) = \inf_{C\in\mathcal{C}} \rho(W_{\mathbf{\Pi}^S(C)};F_1,F_2)$. □

Lemma 4 only uses that the matrices $\mathbf{\Pi}^S(C)$ are symmetric and that $\mathbf{\Pi}$ is a submatrix of $\mathbf{\Pi}^S(C)$; the final rows and final columns of $\mathbf{\Pi}^S(C)$ could potentially be of larger cardinality than the ordered set-theoretic union $\mathbf{\Pi}_{I\cdot}\sqcup\mathbf{\Pi}_{\cdot J}$. The following result shows that our definition of $\mathbf{\Pi}^S(C)$ in eq. (38) is minimal.

Lemma 5. *Let* $\mathbf{\Pi}$ *be a cumulative probability matrix and* $\mathcal{S}$ *denote the set of all symmetric extensions of* $\mathbf{\Pi}$*. Then a matrix* A *is a symmetric extension of* $\mathbf{\Pi}$ *if and only if there is a symmetric copula* C *compatible with* $\mathbf{\Pi}$ *so that* $A = \mathbf{\Pi}^S(C)$*.*

Proof. ($\Rightarrow$) Let A be a symmetric extension of $\mathbf{\Pi}$. We need to show there exists a symmetric copula C so that $A = \mathbf{\Pi}^S(C)$. We will show that M_A is such a copula. Since A is symmetric (Definition 3 (1)), also M_A is a symmetric copula from Lemma 3 (ii). We therefore only need to show that M_A is compatible with $\mathbf{\Pi}$. But this holds using Lemma 3 (iii), since $\mathbf{\Pi}$ is a submatrix of A by Definition 3 (2).

($\Leftarrow$) Let C be compatible with $\mathbf{\Pi}$ and symmetric. Then the $K \times K$ matrix $\mathbf{\Pi}^S(C)$ in eq. (38) satisfies condition (1) and (3) of a symmetric extension by construction. We need to show that $\mathbf{\Pi}$ is a submatrix $\mathbf{\Pi}^S(C)$. That is, we need to show there are row indices $(l_1, l_2, \ldots, I)$ and column indices $(k_1, k_2, \ldots, J)$ so that $\mathbf{\Pi}_{ij} = \mathbf{\Pi}^S(C)_{k_i l_j}$. Let i, j be arbitrary. Since the final row and column of $\mathbf{\Pi}^S(C)$ equals $\mathbf{\Pi}_{I\cdot} \sqcup \mathbf{\Pi}_{\cdot J}$, there is an index l_i so that $\mathbf{\Pi}_{iJ} = \mathbf{\Pi}^S(C)_{l_i K}$, and an index k_j so that $\mathbf{\Pi}_{Ij} = \mathbf{\Pi}^S(C)_{K k_j}$. Since C is compatible with $\mathbf{\Pi}^S(C)$ by definition, we have that $C(\mathbf{\Pi}^S(C)_{l_i K}, \mathbf{\Pi}^S(C)_{K k_j}) = \mathbf{\Pi}^S(C)_{l_i k_j}$. But as C is compatible with $\mathbf{\Pi}$, we also have that $C(\mathbf{\Pi}^S(C)_{l_i K}, \mathbf{\Pi}^S(C)_{K k_j}) = C(\mathbf{\Pi}_{iJ}, \mathbf{\Pi}_{Ij}) = \mathbf{\Pi}_{ij}$. Combining these two equations, we find that $\mathbf{\Pi}^S(C)_{l_i k_j} = \mathbf{\Pi}_{ij}$, hence $\mathbf{\Pi}$ is a submatrix of $\mathbf{\Pi}^S(C)$. □

It immediately follows from the preceding lemma that, when $\mathcal{S}$ is the set of symmetric extensions of $\mathbf{\Pi}$,

$$\begin{aligned} \inf_{A \in \mathcal{S}} \rho(W_A; F_1, F_2) &= \inf_{C \in \mathcal{C}} \rho(W_{\mathbf{\Pi}^S(C)}; F_1, F_2), \\ \sup_{A \in \mathcal{S}} \rho(M_A; F_1, F_2) &= \sup_{C \in \mathcal{C}} \rho(M_{\mathbf{\Pi}^S(C)}; F_1, F_2). \end{aligned} \tag{41}$$

To arrive at the conclusion of Theorem 4 we will need the fact that $\mathcal{S}$ is compact and convex.

Lemma 6. *Let* $\mathbf{\Pi}$ *be a cumulative probability matrix and* $\mathcal{S}$ *denote the set of all symmetric extensions of* $\mathbf{\Pi}$*. The set* $\mathcal{S}$ *is compact and convex.*

Proof. Let A and B be two matrices in $\mathcal{S}$ and $\lambda \in [0, 1]$, and $C = \lambda A + (1 - \lambda)B$. We need to show that conditions (1) - (3) of a symmetric extension (Definition 3) are satisfied. Condition (1) is satisfied since both matrices A and B are symmetric extensions of the same $\mathbf{\Pi}$. Condition (2) also follows, since the condition holds for both matrices A and B. Condition (3) also holds, as a system of linear inequalities form a convex set.

That $\mathcal{S}$ is compact follows from the Heine–Borel theorem, using the topology of $\mathbb{R}^{K^2}$ on $\mathcal{S}$ (which consists of $K \times K$ matrices). The set is bounded, as all its elements are cumulative probability matrices, hence all of their elements are bounded by 1. The set is also closed, as conditions (1), (2) and (3) in Definition 3, are preserved under limits in $\mathbb{R}^{K^2}$, as we show below.

Consider property (1) first; the limit of symmetric matrices is symmetric, and the final rows and columns are kept constant under limits. Similarly, property (2), that $\mathbf{\Pi}$ is a submatrix of $\mathbf{\Pi}^S$, is also preserved under limits. Let $\mathbf{\Pi}_n^S \to \mathbf{\Pi}^S$, and note that $\mathbf{\Pi}$ is a submatrix of $\mathbf{\Pi}_n^S$ at the same indices for each n, thus $\mathbf{\Pi}$ is a submatrix of $\mathbf{\Pi}^S$ too. (This result is also true in general: When B is a submatrix of A_n for all n and $A_n \to A$, B is a submatrix of A as well. But the proof is slightly more involved and is omitted.) Finally, for condition (3), let $\mathbf{\Pi}_n^S$ be a sequence of cumulative probability matrices with limit $\mathbf{\Pi}^S$. Then $(\mathbf{\Pi}_n^S)_{KK} = 1$ for all n, hence $\mathbf{\Pi}_{KK}^S = 1$ as well. Since $\mathbf{\Pi}_n^S$ are cumulative probability

matrices, Corollary 4 shows that $T^{-1}(\mathbf{\Pi}_n^S) \geq 0$. By continuity of T^{-1}, $T^{-1}(\mathbf{\Pi}^S)_{ij} \geq 0$ for all i,j as well, hence $\mathbf{\Pi}^S$ is a cumulative probability matrix by Corollary 4. □

Now we are ready to prove Theorem 4.

Proof of Theorem 4. From the equations (41) and Lemma 6 we find that

$$\begin{aligned}\min_{A\in\mathcal{S}} \rho(W_A; F_1, F_2) &= \min_{C\in\mathcal{C}} \rho(W_{\mathbf{\Pi}^S(C)}; F_1, F_2),\\ \max_{A\in\mathcal{S}} \rho(M_A; F_1, F_2) &= \max_{C\in\mathcal{C}} \rho(M_{\mathbf{\Pi}^S(C)}; F_1, F_2).\end{aligned}$$

Since $\mathcal{S}$ is convex (hence connected) and $A \mapsto \rho(A; F_1, F_2)$ is continuous, the intermediate value theorem tells us that every value between the lower and upper limits are attained, hence

$$\rho_{\mathbf{\Pi}}(\mathcal{F}_{\mathcal{S}}) = [\min_{A\in\mathcal{S}} \rho(W_A; F_1, F_2), \max_{A\in\mathcal{S}} \rho(M_A; F_1, F_2)],$$

as claimed. □

11. Proof of Theorem 1

Proof of Theorem 1. The proof requires the following three steps. (i) We show that the upper and lower copula bounds are copula shuffles with parameters $(u^U, v^U, +)$ for the upper bound and $(u^L, v^L, -)$ for the lower bound, using the definition of a copula shuffle in Definition 1. (ii) We apply Lemma 1 to calculate the correlation bounds. (iii) We use Theorem 3 to demonstrate that the whole partial identification region equals $[L, U]$,where L and U are the correlations obtained in step (ii).

Step (ii) is immediate, and step (iii) holds as explained in Section 9.1 (p. A7). Now we provide details for step (i). From Proposition 2 of Genest and Nešlehová (2007), we know that the upper and lower copula bounds can be written as

$$M_{\mathbf{\Pi}}(u,v) = \sum_{i=1}^{I}\sum_{j=1}^{J} \max\left\{0, \min(u-\alpha_{ij}, v-\beta_{ij}, \pi_{ij})\right\}, \tag{42}$$

$$W_{\mathbf{\Pi}}(u,v) = \sum_{i=1}^{I}\sum_{j=1}^{J} \max\left\{0, -\pi_{ij} + \min(u-\gamma_{ij}, \pi_{ij}) + \min(v-\delta_{ij}, \pi_{ij})\right\}. \tag{43}$$

Here, $\pi_{ij} = P(X=i, Y=j)$ and $\alpha, \beta, \gamma, \delta$ are defined in equation (4) (p. 5).

We need to show $M_{\mathbf{\Pi}} = C_{u^U v^U +}$ and $W_{\mathbf{\Pi}} = C_{u^L v^L -}$, where $C_{uv\pm}$ as defined in Proposition 3 (p. A3). We will only do this for $M_{\mathbf{\Pi}}$, as the other case is virtually identical.

From the definition of C_{uv+}we find that $C_{u^U v^U +}$ of is

$$C_{u^U v^U +}(u,v) = \sum_{k=1}^{KJ} \max\left\{0, \min(u-u_k^U, v-v_k^U, u_{k+1}^U - u_k^U)\right\},$$

which is identical to $M_{\mathbf{\Pi}}$ except for the term $u_{k+1}^U - u_k^U$. To show that $C_{u^U v^U +} = M_{\mathbf{\Pi}}$, we need to show that $u_{k+1}^U - u_k^U$ equals the right π_{ij} after reindexation.

To make this precise, define the pairing function

$$\iota(k) = \begin{cases} (1,k) & 0 < k \leq J,\\ (2, k-J) & J < k \leq 2J,\\ \vdots & \vdots \\ (I, k-(I-1)J) & (I-1)J < k \leq IJ.\end{cases}$$

Then $u_k^U = \alpha_{\iota(k)}$ if $k \leq IJ$ and 1 if $k = IJ+1$ while $v_k^U = \beta_{\iota(k)}$ for all k.

We need to show that $u^U_{k+1} - u^U_k = \pi_{\iota(k)}$. To do so, first assume that $\iota(k) = (i, j)$ with $j < J$. Then $\iota(k+1) = (i, j+1)$, hence

$$\begin{aligned}
u^U_{k+1} - u^U_k &= [\mathbf{\Pi}_{(i-1)J} + \mathbf{\Pi}_{ij} - \mathbf{\Pi}_{(i-1)}] - [\mathbf{\Pi}_{(i-1)J} + \mathbf{\Pi}_{i(j-1)} - \mathbf{\Pi}_{(i-1)(j-1)}],\\
&= [\mathbf{\Pi}_{ij} - \mathbf{\Pi}_{(i-1)j}] - [\mathbf{\Pi}_{i(j-1)} - \mathbf{\Pi}_{(i-1)(j-1)}],\\
&= \pi_{ij} = \pi_{\iota(k)}.
\end{aligned}$$

On the other hand, if $\iota(k) = (i, j)$ with $j = J$ and $i < I$, then $\iota(k+1) = (i+1, 1)$, hence

$$\begin{aligned}
u^U_{k+1} - u^U_k &= \mathbf{\Pi}_{iJ} - [\mathbf{\Pi}_{(i-1)J} + \mathbf{\Pi}_{i(J-1)} - \mathbf{\Pi}_{(i-1)(J-1)}],\\
&= [\mathbf{\Pi}_{iJ} - \mathbf{\Pi}_{(i-1)J}] - [\mathbf{\Pi}_{i(J-1)} - \mathbf{\Pi}_{(i-1)(J-1)}],\\
&= \pi_{iJ} = \pi_{\iota(k)}.
\end{aligned}$$

Finally, if if $\iota(k) = (i, j)$ with $j = J$ and $i = I$, then

$$\begin{aligned}
u^U_{k+1} - u^U_k &= \mathbf{\Pi}_{IJ} - [\mathbf{\Pi}_{(I-1)J} + \mathbf{\Pi}_{I(J-1)} - \mathbf{\Pi}_{(I-1)(J-1)}],\\
&= \pi_{IJ} = \pi_{\iota(k)}.
\end{aligned}$$

□

12. Proof of Corollary 1.

Proof of Corollary 1. From the Höffding (1940) formula for covariance reproduced in eq. (11), we may write $\rho_L = \rho(W_{\mathbf{\Pi}}; F_1, F_2)$ and $\rho_U = \rho(M_{\mathbf{\Pi}}; F_1, F_2)$ using the formulas for $W_{\mathbf{\Pi}}$ and $M_{\mathbf{\Pi}}$ from Section 9.1. Proposition 6 in Genest and Nešlehová (2007) implies that both $M_{\mathbf{\Pi}^n}$ and $W_{\mathbf{\Pi}^n}$ converge to C uniformly, and therefore also pointwise, as long as the X- and Y-mesh of $(\mathbf{\Pi}^n)$ decreases uniformly to zero. The dominated convergence theorem (with the classical Fréchet–Höffding bounds as dominating functions) therefore implies that both $\rho(M_{\mathbf{\Pi}^n}; F_1, F_2)$ and $\rho(M_{\mathbf{\Pi}^n}; F_1, F_2)$ converge to $\rho(C; F_1, F_2)$ using the formula in eq. (11). Therefore the two endpoints of $\rho_{\mathbf{\Pi}^n}(\mathcal{F})$ both converge to $\rho(C; F_1, F_2)$. □

13. Proof of Theorem 2

Proof of Theorem 2. By Proposition 1 in Foldnes and Grønneberg (2019a), there exists a continuous random vector $Z = (Z_1, Z_2)$ that generates the ordinal (X, Y) through eq. (1). Then the copula C of Z will also generate $\mathbf{\Pi}^\star$, i.e., $\mathbf{\Pi}^\star_{iv} = C(\mathbf{\Pi}^\star_{i1}, \mathbf{\Pi}^\star_{I1}) = C(\mathbf{\Pi}^\star_{i1}, v)$. Since both $\{\mathbf{\Pi}_{i1}\}_{i=1}^I$ and $[0, 1]$ are compact, we may use Proposition 4 with $\mathcal{S} = \{\mathbf{\Pi}_{i1}\}_{i=1}^I \times [0, 1]$ and the copula C to deduce the limit copulas

$$\begin{aligned}
M_{\mathbf{\Pi}^\star}(u, v) &= \min\left\{u, v, \min_{1\le i\le I-1}\min_{0\le z\le 1}[\mathbf{\Pi}^\star_{iz} + (u - \mathbf{\Pi}^\star_{i1})^+ + (v - z)^+]\right\},\\
W_{\mathbf{\Pi}^\star}(u, v) &= \max\left\{0, u + v - 1, \max_{1\le i\le I-1}\max_{0\le z\le 1}[\mathbf{\Pi}^\star_{iz} - (\mathbf{\Pi}^\star_{i1} - u)^+ - (z - v)^+]\right\}.
\end{aligned}$$

These copulas are not on the form given in the statement of this Theorem. But following the same argument as in the proof of Lemma 2 in Grønneberg et al. (2020), we find that the inner minima and maxima can be written as

$$\min_{0\le z\le 1}[\mathbf{\Pi}^\star_{iz} + (u - \mathbf{\Pi}^\star_{i1})^+ + (v - z)^+] = \mathbf{\Pi}^\star_{iv} + (u - \mathbf{\Pi}^\star_{i1})^+$$
$$\max_{0\le z\le 1}[\mathbf{\Pi}^\star_{iz} - (\mathbf{\Pi}^\star_{i1} - u)^+ - (z - v)^+] = \mathbf{\Pi}^\star_{iv} - (\mathbf{\Pi}^\star_{i1} - u)^+.$$

Plugging these results into the equations for $M_{\mathbf{\Pi}^\star}$ and $W_{\mathbf{\Pi}^\star}$ above, we end up with

$$M_{\mathbf{\Pi}^\star}(u,v) = \min(u, v, \min_{1\leq i\leq I-1}(\mathbf{\Pi}^\star_{iv} + (u - \mathbf{\Pi}^\star_{i1})^+),$$
$$W_{\mathbf{\Pi}^\star}(u,v) = \max(0, u+v-1, \max_{1\leq i\leq I-1}(\mathbf{\Pi}^\star_{iv} - (\mathbf{\Pi}^\star_{i1} - u)^+).$$

The result then follows from an application of Theorem 3. □

14. Proof of Corollary 2

Proof of Corollary 2. Our proof consists of showing the pointwise convergence of $W_{\mathbf{\Pi}^n}, M_{\mathbf{\Pi}^n}$ to $M_{\mathbf{\Pi}^\star}, W_{\mathbf{\Pi}^\star}$ as $n \to \infty$. The proof then proceeds as in Corollary 1, and is omitted. We restrict attention to the relationship between the lower bounds, as the argument for the upper bounds follows by the same steps.

Following the same steps as in the simplification derived in Section 9.1, we see that

$$W_{\mathbf{\Pi}^\star}(u,v) = \max\left\{0, u+v-1, \max_{i\in\{i(u), i(u)+1\}\setminus\{0,I\}}[C(\mathbf{\Pi}^\star_{i1}, v) - (\mathbf{\Pi}^\star_{i1} - u)^+]\right\} \tag{44}$$

where $i(u)$ is the largest index $1 \leq i \leq I$ satisfying $P(X_n \leq i) < u$.

For a $v \in [0,1]$, define $j_n(v)$ as the largest index $1 \leq j \leq J_n$ satisfying $\mathbf{\Pi}_{Ij} < u$. Recall that $(\mathbf{\Pi}^n_{Ij})_{j=0}^{J_n}$ is an increasing sequence in $[0,1]$ with the endpoints $\mathbf{\Pi}^n_{I0} = 0, \mathbf{\Pi}^n_{IJ_n} = 1$ included, and where the maximum difference between its consecutive elements go to zero. The sequence $(\mathbf{\Pi}^n_{Ij})_{j=0}^{J_n}$ therefore eventually (as $n \to \infty$) approximate any number $v \in [0,1]$ with arbitrary precision. Therefore, for any point $v \in [0,1]$, we have that

$$\lim_{n\to\infty} \mathbf{\Pi}^n_{Ij_n(v)} = v, \quad \lim_{n\to\infty} \mathbf{\Pi}^n_{I[j_n(v)+1]} = v. \tag{45}$$

Using eq. (31) and eq. (32) on p. A8, we get that for each $0 \leq u, v \leq 1$

$$\begin{aligned}
W_{\mathbf{\Pi}^n}(u,v) &= \max\Bigg(0, u+v-1, \max_{i\in\{i(u),i(u)+1\}\setminus\{0,I\}} \max_{j\in\{j_n(v),j_n(v)+1\}\setminus\{0,J_n\}} \\
&\qquad [\mathbf{\Pi}^n_{ij} - (\mathbf{\Pi}^n_{iJ_n} - u)^+ - (\mathbf{\Pi}^n_{Ij} - v)^+]\Bigg) \\
&= \max\Bigg(0, u+v-1, \max_{i\in\{i(u),i(u)+1\}\setminus\{0,I\}} \max_{j\in\{j_n(v),j_n(v)+1\}\setminus\{0,J_n\}} \\
&\qquad [C(\mathbf{\Pi}^n_{iJ_n}, \mathbf{\Pi}^n_{Ij}) - (\mathbf{\Pi}^n_{iJ_n} - u)^+ - (\mathbf{\Pi}^n_{Ij} - v)^+]\Bigg) \\
&\overset{(a)}{=} \max\Bigg(0, u+v-1, \max_{i\in\{i(u),i(u)+1\}\setminus\{0,I\}} \max_{j\in\{j_n(v),j_n(v)+1\}\setminus\{0,J_n\}} \\
&\qquad [C(\mathbf{\Pi}^\star_{i1}, \mathbf{\Pi}^n_{Ij}) - (\mathbf{\Pi}^\star_{i1} - u)^+ - (\mathbf{\Pi}^n_{Ij} - v)^+]\Bigg) \\
&\xrightarrow[n\to\infty]{(b)} \max\Bigg(0, u+v-1, \max_{i\in\{i(u),i(u)+1\}\setminus\{0,I\}} \\
&\qquad [C(\mathbf{\Pi}^\star_{i1}, v) - (\mathbf{\Pi}^\star_{i1} - u)^+ - (v - v)^+]\Bigg) \\
&\overset{(c)}{=} W_{\mathbf{\Pi}^\star}(u,v).
\end{aligned}$$

(Step a). We have $\mathbf{\Pi}^n_{iJ_n} = \mathbf{\Pi}^\star_{i1}$, which can be seen as follows. Notice that $P(X \leq i) = P(X \leq i, y \leq 1) = \mathbf{\Pi}^\star_{i1}$, and that $P(X \leq i) = P(X \leq i, Y_n \leq J_n) = \mathbf{\Pi}^n_{iJ_n}$, where $Y_n = \sum_{j=1}^{J_n} jI\{\mathbf{\Pi}^n_{I(j-1)} < F_2(Z_2) \leq \mathbf{\Pi}^n_{Ij}\}$.

(Step b). The index j appears only in the context of $\mathbf{\Pi}^n_{Ij}$, and j is either $j = j_n(v)$ or $j = j_n(v)+1$. From eq. (45) we have $\mathbf{\Pi}^n_{Ij_n(v)} \to v$ and $\mathbf{\Pi}^n_{I[j_n(v)+1]} \to v$. Since maximums are continuous, and C is a copula and hence uniformly continuous from the Lipschitz condition, we get the convergence.

(Step c). Use eq. (44).

□

15. Proof of Lemma 2

Proof of Lemma 2. (i) For all $(u, v) \in \mathcal{S}$, we have $\lambda D(u, v)+(1-\lambda)E(u, v) = \lambda C(u, v)+(1-\lambda)C(u, v) = C(u, v)$. Therefore, $C^\lambda(u, v)$ agrees with C on $\mathcal{S}$. (iii) It is well known that a convex combination of copulas is a copula. See e.g. exercise 2.3 and 2.4 of Nelsen (2007). (ii) We only need to verify the Lipschitz inequality, as the other defining properties of a quasi-copula are immediately verified. That C^λ is a quasi-copula follows from the triangle inequality, as

$$\begin{aligned}
&|\lambda E(u_2, v_2) + (1-\lambda)D(u_2, v_2) - [\lambda E(u_1, v_1) + (1-\lambda)D(u_1, v_1)]|, \\
\leq\ &\lambda|E(u_2, v_2) - E(u_1, v_1)| + (1-\lambda)|D(u_2, v_2) - D(u_1, v_1)| \\
\leq\ &\lambda(|u_2 - u_1| + |v_2 - v_1|) + (1-\lambda)||u_2 - u_1| + |v_2 - v_1|| \\
=\ &|u_2 - u_1| + |v_2 - v_1|.
\end{aligned}$$

□

16. Proof of Lemma 7, used in Proposition 4

Lemma 7. *Let $\mathcal{S} \subseteq \mathcal{S}_1 \times \mathcal{S}_2$ for some sets $\mathcal{S}_1, \mathcal{S}_2$. Then the following are equivalent.*

(1) If $(a_1, b_1) \in \mathcal{S}$ and $(a_2, b_2) \in \mathcal{S}$, then $(a_1, b_2) \in \mathcal{S}$ and $(a_2, b_1) \in \mathcal{S}$.
(2) There are sets A, B so that $\mathcal{S} = A \times B$.

Proof. Suppose (1) is true. Define $A = \{a : (a, b) \in \mathcal{S} \text{ for some } b \in \mathcal{S}_2\}$ and $B = \{b : (a, b) \in \mathcal{S} \text{ for some } a \in \mathcal{S}_1\}$. We have $\mathcal{S} \subseteq A \times B$, since $A \times B = \{(a, b) : a \in A, b \in B\}$ consists of all pairs where the coordinate of each element can occur in $\mathcal{S}$. That is, if $(a, b) \in \mathcal{S}$, then $a \in A$ and $b \in B$, and therefore $(a, b) \in A \times B$.

We also have $A \times B \subseteq \mathcal{S}$. To see this, notice that if $(a, b) \in A \times B$ we know by the definition of A, B that there are b' and a' such that $(a, b') \in \mathcal{S}$ and $(a', b) \in \mathcal{S}$. By the assumption on $\mathcal{S}$, with $a_1 = a, b_1 = b', a_2 = a', b_2 = b$ we conclude that both $(a_1, b_2) = (a, b) \in \mathcal{S}$ and $(a_2, b_1) = (a', b') \in \mathcal{S}$. From the first conclusion, we have $(a, b) \in \mathcal{S}$, so that $A \times B \subseteq \mathcal{S}$, since (a, b) were arbitrary elements in $\mathcal{S}$. We conclude that $\mathcal{S} = A \times B$.

Suppose (2) is true. We have $\mathcal{S} = A \times B = \{(a, b) : a \in A, b \in B\}$. Suppose $(a_1, b_1) \in \mathcal{S}$ and $(a_2, b_2) \in \mathcal{S}$. This means $a_1, a_2 \in A, b_1, b_2 \in B$. Therefore $(a_1, b_2) \in A \times B$ by definition of $A \times B$, and also $(a_2, b_1) \in A \times B$. □

17. Proof of Proposition 2 in Appendix 10.1

Proof of Proposition 2. If $\mathbf{\Pi}$ is a cumulative probability matrix there is a copula C compatible with it. The copula is two-decreasing (see equation (13) on page A1 of the main document), hence (12) holds. The other way around follows from Corollary 4 below. □

Define the linear operator $T : \mathbb{R}^{K\times L} \to \mathbb{R}^{K\times L}$ by

$$(TA)_{ij} = \sum_{k=1}^{i}\sum_{l=1}^{j} A_{kl} \tag{46}$$

The rôle of this operator is to translate potential probability matrices (matrices with non-zero elements summing to 1) into potential cumulative probability matrices $\mathbf{\Pi}$. We may also go in the opposite direction, as shown by the following Lemma.

Lemma 8. *The operator T is invertible. Its inverse $T^{-1} = S$ is defined by*

$$(SB)_{ij} = B_{ij} - B_{(i-1)j} - B_{i(j-1)} + B_{(i-1)(j-1)}, \quad 1 \le i \le K, 1 \le j \le L, \tag{47}$$

where as a short-hand we write $B_{0j} = B_{i0} = 0$.

Proof. It suffices to show that $STA = A$ for any matrix A. That is, to show that S is the left-inverse of T. From its definition and the linearity of sums, it is seen that T is a linear operator on $\mathbb{R}^{KL}$. Therefore, T can be regarded as a square $KL \times KL$ matrix. Since it is well-known that left-inverses and right-inverses coincide for matrices, we may conclude that $ST = TS = I$ and that S is the unique inverse of T once we have shown it to be a left-inverse. Let $1 \le i \le K, 1 \le j \le L$, and use the convention that sums over empty sets are zero. We then have

$$\begin{aligned}
(STA)_{ij} &= (TA)_{ij} - (TA)_{(i-1)j} - (TA)_{i(j-1)} + (TA)_{(i-1)(j-1)},\\
&= \sum_{k=1}^{i}\sum_{l=1}^{j} A_{kl} - \sum_{k=1}^{i-1}\sum_{l=1}^{j} A_{kl} - \sum_{k=1}^{i}\sum_{l=1}^{j-1} A_{kl} + \sum_{k=1}^{i-1}\sum_{l=1}^{j-1} A_{kl},\\
&= \left[\sum_{k=1}^{j}\sum_{l=1}^{i} A_{kl} - \sum_{k=1}^{j}\sum_{l=1}^{i-1} A_{kl}\right] - \left[\sum_{k=1}^{j-1}\sum_{l=1}^{i} A_{kl} - \sum_{k=1}^{j-1}\sum_{l=1}^{i-1} A_{kl}\right],\\
&= \sum_{k=1}^{j}\left[\sum_{l=1}^{i} A_{kl} - \sum_{l=1}^{i-1} A_{kl}\right] - \sum_{k=1}^{j-1}\left[\sum_{l=1}^{i} A_{kl} - \sum_{l=1}^{i-1} A_{kl}\right],\\
&= \sum_{k=1}^{j} A_{ik} - \sum_{k=1}^{j-1} A_{ik}.
\end{aligned}$$

Since $\sum_{k=1}^{j} A_{ik} - \sum_{k=1}^{j-1} A_{ik} = A_{ij}$, we find that $(STA)_{ij} = A_{ij}$, as claimed. These calculations also hold if one or more of the sums are zero, as they are when $i = 1$ or $j = 1$. □

Corollary 4. *Let A be a $K \times L$ matrix. For $1 \le k \le K, 1 \le j \le L$, we have*

$$\sum_{i=1}^{k}\sum_{j=1}^{l} T^{-1}(A)_{ij} = A_{kl}.$$

Moreover, A is a cumulative probability matrix if and only if both $A_{KL} = 1$ and $T^{-1}(A)_{ij} \ge 0$ for all i, j

Proof. We know that $T(B)_{kl} = \sum_{i=1}^{k}\sum_{j=1}^{l} B_{ij}$ by definition, hence

$$\sum_{i=1}^{k}\sum_{j=1}^{l}\left[T^{-1}(A)_{ij}\right] = (TT^{-1}A)_{kl} = A_{kl}.$$

This also holds when $i = K, j = L$, hence $A_{KL} = 1$ is equivalent to

$$\sum_{i=1}^{K}\sum_{j=1}^{L}\left[T^{-1}(A)_{ij}\right] = 1.$$

For the second statement, we recall that a probability mass function is characterized by having elements in $[0,1]$ and sum to one. Then it induces a valid probability distribution.

Assume that $T^{-1}(A)_{ij} \geq 0$ for all i, j and $A_{KL} = 1$. Then all elements of $T^{-1}(A)$ are non-negative and its elements sum to 1 by the equation above. Since the sum of non-negative terms is greater than or equal to its largest term, we get that also $T^{-1}(A)_{ij} \leq 1$. The matrix $T^{-1}(A)$ therefore has elements in $[0,1]$ and sum to one, and is therefore a valid probability matrix.

Next, assume that $T^{-1}(A)$ is a probability matrix. By definition, we have $0 \leq T^{-1}(A)_{ij} \leq 1$, which implies that $T^{-1}(A)_{ij} \geq 0$, for all i, j. By definition, we also know that the elements of $T^{-1}(A)$ sum to 1, which means $1 = \sum_{i=1}^{K}\sum_{j=1}^{L}\left[T^{-1}(A)_{ij}\right] = A_{KL}$, and the conclusion follows. □

References

Bernard, C., Liu, Y., MacGillivray, N., & Zhang, J. (2013). Bounds on capital requirements for bivariate risk with given marginals and partial information on the dependence. *Dependence Modeling*, *1*(2013), 37–53. https://doi.org/10.2478/demo-2013-0002

Carley, H. (2002). Maximum and minimum extensions of finite subcopulas. *Communications in Statistics - Theory and Methods*, *31*(12), 2151–2166. https://doi.org/10.1081/STA-120017218

Foldnes, N., & Grønneberg, S. (2019). On identification and non-normal simulation in ordinal covariance and item response models. *Psychometrika*, *84*(4), 1000–1017. https://doi.org/10.1007/s11336-019-09688-z

Foldnes, N., & Grønneberg, S. (2021). The sensitivity of structural equation modeling with ordinal data to underlying non-normality and observed distributional forms. *Psychological Methods*. https://doi.org/10.1037/met0000385

Genest, C., & Nešlehová, J. (2007). A primer on copulas for count data. *ASTIN Bulletin: The Journal of the IAA*, *37*(2), 475–515. https://doi.org/10.1017/S0515036100014963

Grønneberg, S., Moss, J., & Foldnes, N. (2020). Partial identification of latent correlations with binary data. *Psychometrika*, *85*(4), 1028–1051. https://doi.org/10.1007/s11336-020-09737-y

Höffding, W. (1940). *Maßstabinvariante korrelationstheorie für diskontinuierliche verteilungen* (Unpublished doctoral dissertation). Universität Berlin.

Li, B., & Genton, M. (2013). Nonparametric identification of copula structures. *Journal of the American Statistical Association*, *108*(502), 666–675. https://doi.org/10.1080/01621459.2013.787083

Mikusinski, P., Sherwood, H., & Taylor, M. D. (1992). Shuffles of min. *Stochastica*, *13*(1), 61–74.

Nelsen, R. B. (1993). Some concepts of bivariate symmetry. *Journal of Nonparametric Statistics*, *3*(1), 95–101. https://doi.org/10.1080/10485259308832574

Nelsen, R. B. (2007). *An introduction to copulas*. Springer Science & Business Media. https://doi.org/10.1007/978-1-4757-3076-0

Olsson, U. (1979). Maximum likelihood estimation of the polychoric correlation coefficient. *Psychometrika*, *44*(4), 443–460. https://doi.org/10.1007/BF02296207

Sklar, M. (1959). Fonctions de répartition à n dimensions et leurs marges. *Publ. Inst. Statist. Univ. Paris 8*, 229–231.

Tankov, P. (2011). Improved Fréchet bounds and model-free pricing of multi-asset options. *Journal of Applied Probability*, *48*(2), 389–403. https://doi.org/10.1239/jap/1308662634

Department of Data Science and Analytics, BI Norwegian Business School, Oslo, Norway 0484
Email address: jonas.moss.statistics@gmail.com

Department of Economics, BI Norwegian Business School, Oslo, Norway 0484
Email address: steffeng@gmail.com
