# Supplementary figures and images for: Partial Identification of Latent Correlations with Ordinal Data
Source: Psychometrika. 2023 Jan 31;88(1):241–52. doi: 10.1007/s11336-022-09898-y (PMC9977897; doi:10.1007/s11336-022-09898-y)

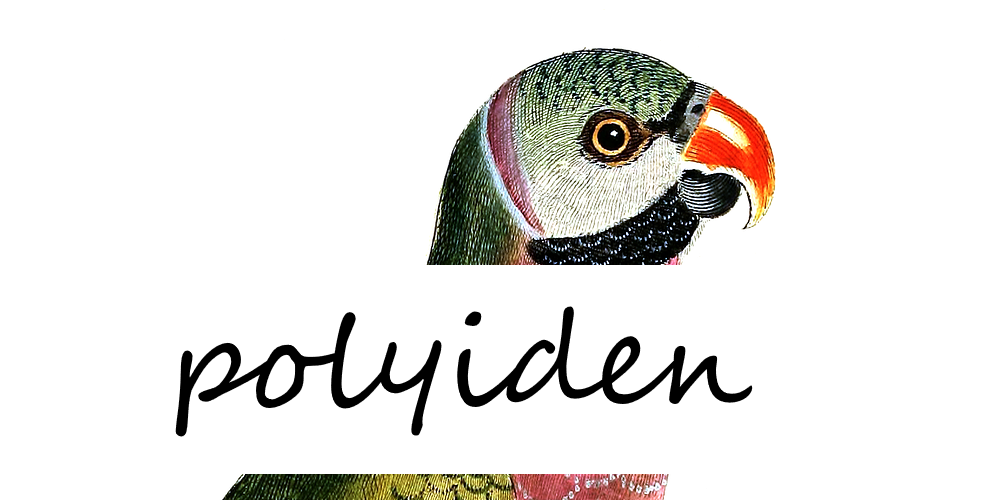

Supplement: Supplementary file 12 — (zip 1097 KB) [file 11336_2022_9898_MOESM12_ESM.zip › man/figures/logo.png]
